# A stable isotope dilution tandem mass spectrometry method of major kavalactones and its applications

**DOI:** 10.1371/journal.pone.0197940

**Published:** 2018-05-24

**Authors:** Yi Wang, Shainnel O. Eans, Heather M. Stacy, Sreekanth C. Narayanapillai, Abhisheak Sharma, Naomi Fujioka, Linda Haddad, Jay McLaughlin, Bonnie A. Avery, Chengguo Xing

**Affiliations:** 1 Department of Medicinal Chemistry, University of Florida, Gainesville, Florida, United States of America; 2 Department of Pharmacodynamics, University of Florida, Gainesville, Florida, United States of America; 3 Department of Medicinal Chemistry, University of Minnesota, Minneapolis, Minnesota, United States of America; 4 Department of Pharmaceutics, University of Florida, Gainesville, Florida, United States of America; 5 Masonic Cancer Center, University of Minnesota, Minneapolis, Minnesota, United States of America; 6 Department of Family Community and Health System Sciences, University of Florida, Gainesville, Florida, United States of America; University of Pittsburgh, UNITED STATES

## Abstract

Kava is regaining its popularity with detailed characterizations warranted. We developed an ultraperformance liquid chromatography high-resolution tandem mass spectrometry (UPLC-MS/MS) method for major kavalactones (kavain, dihydrokavain, methysticin, dihydromethysticin and desmethoxyyangonin) with excellent selectivity and specificity. The method has been validated for different matrices following the Food and Drug Administration guidance of analytical procedures and methods validation. The scope of this method has been demonstrated by quantifying these kavalactones in two kava products, characterizing their tissue distribution and pharmacokinetics in mice, and detecting their presence in human urines and plasmas upon kava intake. As expected, the abundances of these kavalactones differed significantly in kava products. All of them exhibited a large volume of distribution with extensive tissue affinity and adequate mean residence time (MRT) in mice. This method also successfully quantified these kavalactones in human body fluids upon kava consumption at the recommended human dose. This UPLC-MS/MS method therefore can be used to characterize kava products and its pharmacokinetics in animals and in humans.

## Introduction

Kava is a beverage in the South Pacific regions. It has been documented to help people relax, socialize and improve the quality of sleep [1]. The traditional form of kava is prepared by grinding the rhizome of kava (*Piper methysticum* Forst) in ambient temperature water or coconut milk. Kava can also be prepared by extracting the rhizomes with ethanol or acetone. A number of clinical studies suggest that kava has an anxiolytic effect with the organic extract preparation once marketed as an anxiolytic agent [2–4]. The organic extract form has also been commercialized as a dietary supplement and recent data indicate kava resurgence outside of the South Pacific regions over the past few years [5].

**Fig 1:**
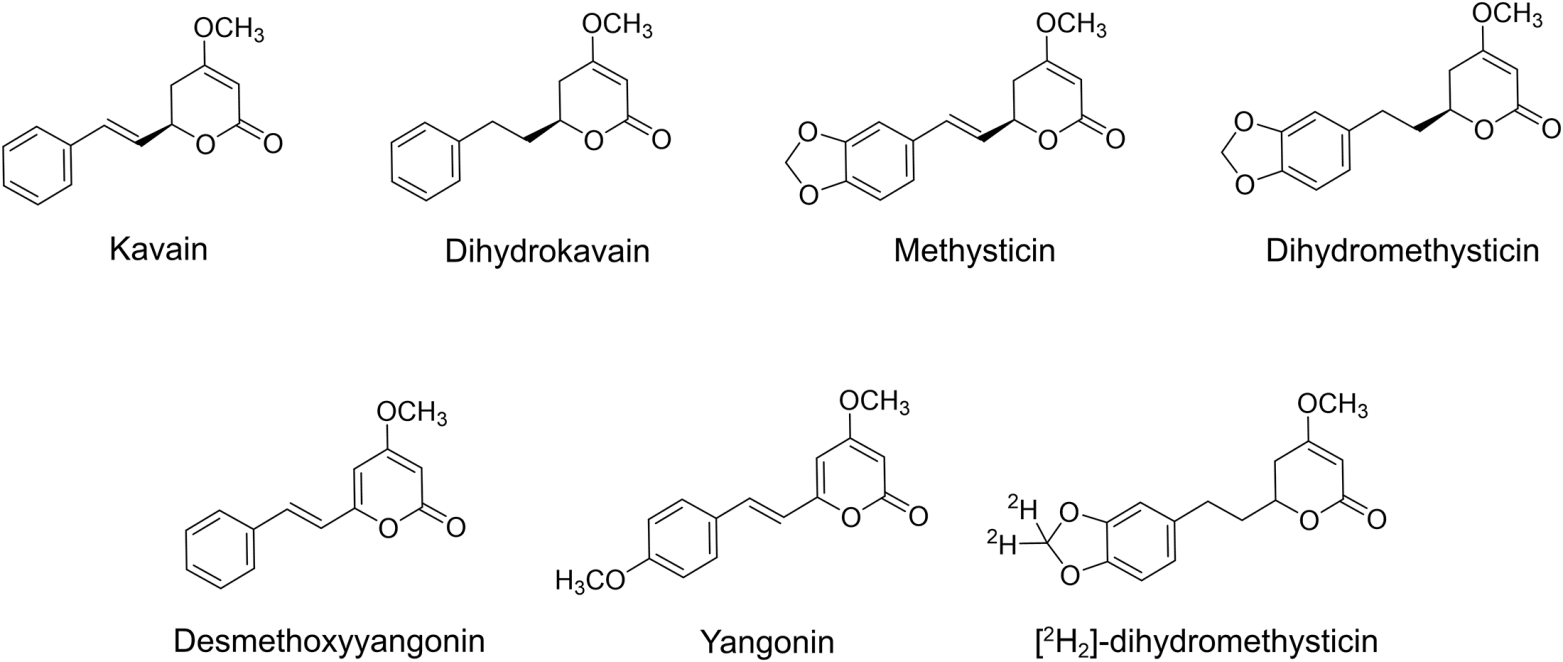
Structures of six major kavalactones in kava and [^2^H_2_]-dihydromethysticin.

Kava contains a set of structurally unique lactones that are dominantly detected in kava, named kavalactones. Six of them have been reported as the major ones, including kavain, dihydrokavain (DHK), methysticin, dihydromethysticin (DHM), desmethoxyyangonin and yangonin (Fig 1) [6]. These kavalactones are generally thought to be the ingredients responsible for its relaxing effect and their total abundance has been used for kava dosing standardization [7]. On the other hand, these kavalactones may provide different contributions to kava’s biological activities in spite of their high structural similarity [8–13]. For instance, two *in vivo* studies suggested that DHK might be more anxiolytic [10, 11], although kavain has been traditionally considered as the major anxiolytic ingredient [12]. These kavalactones may also influence their pharmacokinetics when used together [14–16]. For example, the bioavailability of kavalactones generally increased when being administered in the kava matrix in comparison to being administered alone [14, 15]. Therefore, to better understand their functions, the individual kavalactones in kava products need be quantified in addition to their total abundance.

Beyond potential benefits, reports of rare but sometimes severe hepatotoxic cases among kava users in the late 1990s have brought the public attention to its safety [2], resulting in the ban of kava in Germany between 2001–2014 [17]. The US Food and Drug Administration (FDA) also advised consumers of its hepatotoxic risk in 2002. There has been no further action from FDA, likely because of the lack of solid evidence of kava’s hepatotoxic risk [18, 19]. Although a number of potential causes and mechanisms have been proposed [18, 20], there have been limited investigations to test these hypotheses [21]. One plausible cause is the overdosing with kava prepared from low-quality raw materials [22] and the an organic extraction preparation instead of the traditional aqueous suspension [23]. In order to address the safety concerns of kava, the chemical composition of the commercial kava products needs to be thoroughly profiled as well.

In addition, there have been very few pharmacokinetic characterizations of kava, even in animal models [14, 24, 25], which were performed at dosages significantly higher than the human relevant exposure [26, 27]. Indeed, only one pharmacokinetic study in humans has ever been reported, which used kavain at a dose of 800 mg, much higher than the recommended dose for human use (which is 200–300 mg/person daily) [28]. The lack of the pharmacokinetic knowledge of kava is likely due to the low sensitivity and specificity of its analytical methods, including near-infrared reflectance spectroscopy, high performance thin layer chromatography (HPTLC), high performance liquid chromatography (HPLC), liquid chromatography–mass spectrometry (LC-MS), and nuclear magnetic resonance spectroscopy (NMR) [5, 7, 29–35].

To address these issues, we have developed an ultraperformance liquid chromatography tandem mass spectrometry (UPLC-MS/MS) coupled with the high-resolution Orbitrap, which minimizes the isobaric interferences from the matrix, resulting in increased sensitivity and specificity [36, 37]. The method was validated according to the FDA’s guideline for kavain, DHK, methysticin, DHM and desmethoxyyangonin using a deuterium-labeled DHM (^2^H_2_-DHM, Fig 1) as an internal standard. Yangonin was not included in this study because of its low abundance in the kava products used in this study [38]. This method could quantify these kavalactones in different kava products. The same method was able to characterize the pharmacokinetics and biodistribution of these major kavalactones in C57BL/6J mice at a human relevant kava dose. Lastly, this method successfully quantified these kavalactones in human urines and plasmas after human subjects consumed kava at the recommended dose. These results demonstrate the scope of this UPLC-MS/MS based method.

## Materials and methods

### Chemicals and materials

One kava product in the ethanolic extract format (standardized to 150 mg/mL total kavalactones) and one in the soft-gel capsule format (75 mg total kavalactone per capsule) were purchased from Gaia Herbs, Inc. (Brevard, NC). Kavain, DHK, methysticin, DHM and desmethoxyyangonin were isolated from the ethanolic kava with their structures confirmed via NMR and mass spectrometry [39]. A deuterium labeled dihydromethysticin (^2^H_2_-DHM) was synthesized following our published procedures with slight modifications [40]. LC-MS grade water, formic acid, methanol and acetonitrile were purchased from Sigma-Aldrich (St. Louis, MO). All other chemicals were ACS grade unless stated otherwise. SOLA HRP solid-phase extraction (SPE) cartridges (10 mg) were purchased from Fisher Scientific (Rockford, IL).

### UPLC-MS/MS method validation and calibration curves

Mass spectra of the five kavalactones were first acquired by direct infusion of the pure compounds. UPLC-MS/MS was performed with a Dionex Ultimate 3000 RS and a Q Exactive Hybrid Quadrupole Orbitrap Mass Spectrometer (Thermo Fisher Scientific, San Jose, CA). Briefly, the samples (5 *μ*L) were resolved through an Atlantis dc18 column (150 x 2.1 mm, 3 *μ*m particle size, 100 Å) with a 25 min linear gradient from 99% A (H_2_O with 1% CH_3_CN and 0.05% HCO_2_H) to 99% B (CH_3_CN with 5% H_2_O and 0.05% HCO_2_H) at a flow rate of 250 *μ*L/min. The parameters for Heated Electrospray Ionization (HESI-II) were set as follows: sheath gas, 50; auxiliary gas, 15; auxiliary gas temperature, 300 °C; capillary temperature, 300 °C; spray voltage, 4 kV; 1 *μ*scan; maximum injection time, 200 ms for MS/MS; HCD, 20 for all kavalactones. Resolution was set as 17,500 at *m/z* 200 for MS/MS. The isolation width was set at *m*/*z* 1 for MS/MS scan modes. AGC (automated gain control) was set at 50,000 for Orbitrap (FT) MS/MS. The method was validated based on the Food and Drug Administration (FDA) Guidance [41]. For the mouse study, selectivity, accuracy, and within-day and between-day precision of the method was validated using mouse serum or tissues (liver, lung and brain) of the control group. For the tissues, kavalactones were spiked at the level of 5, 15, 50 and 90 pg/mg tissue. For mouse serum, kavalactones were spiked at the level of 0.8, 8, 80 and 8000 pg/*μ*L serum. For the human study, the method was validated based on the accuracy, within-day and between-day precision using the pre-kava urine or plasma sample with each kavalactone added at a level of 0.15, 0.45, 1 and 2 pg/*μ*L urine/plasma. The reproducibility studies were based on six independent measurements on three different days. The accuracy and percent coefficient of variation (CV%) were used as the criteria for the precision and reproducibility of the method.

For the quantification of kavalactones in kava products, a seven-point calibration curve was constructed with DHM in 10% CH_3_OH (0, 0.05, 0.10, 0.50, 1.00, 2.50 and 5.00 pg/*μ*L). [^2^H_2_]-DHM was added at a level of 2.50 pg/*μ*L as the internal standard. DHM and [^2^H_2_]-DHM were measured at the MS/MS scan stage using product ions at *m/z* 277.1 > 131.0490, 135.0438, 161.0593 and *m/z* 279.1 > 131.0490, 137.0564, 163.0719 at a mass accuracy window of ± 5 ppm, respectively. The amounts of the other four kavalactones were estimated using the DHM calibration curves but corrected by the ratio of ion peak areas of each kavalactone to DHM (S1 Fig). For the quantitative analyses of kavalactones in mouse samples, a ten-point calibration curve of DHM was constructed (0, 2.5, 5, 10, 50, 150, 250, 500, 2500, and 5000 pg/mg tissue) with [^2^H_2_]-DHM (50 pg/mg tissue or 100 pg/μL serum) as the internal standard. For human samples, a seven-point calibration curve of DHM was constructed (0, 0.05, 0.10, 0.20, 0.50, 1.00, and 2.00 pg/*μ*L sample) with [^2^H_2_]-DHM (1 pg/*μ*L) as the internal standard.

### Profiling five kavalactones in two kava products

The ethanolic kava product was dried under vacuum to remove the solvent, resulting in an oil. The oil product was dissolved in dimethyl sulfoxide (DMSO) to make a kava stock solution (1 mg/mL) and diluted to a final concentration of 10 pg/*μ*L in 10% CH_3_OH in H_2_O with [^2^H_2_]-DHM as the internal standard at a level of 2.5 pg/*μ*L for UPLC-MS/MS analysis. For the kava soft-gel capsule product, all materials in a capsule were dissolved in DMSO to make a stock solution (1 mg/mL) and diluted to a final concentration of 10 pg/*μ*L in 10% CH_3_OH in H_2_O with [^2^H_2_]-DHM at a level of 2.5 pg/*μ*L for UPLC-MS/MS analysis.

### Pharmacokinetic analyses in mice

All mice were housed, tested, and cared for in accordance with the 2011 National Institutes of Health Guide for the Care and Use of Laboratory Animals, and handled according to the animal welfare protocols approved by Institutional Animal Care and Use Committee at the University of Florida. All experiments were carried out using male C57BL/6J mice (The Jackson Laboratory, Bar Harbor, ME) of 10 weeks old. Mice were kept in groups of five in a temperature-controlled room with 12-hour light/dark cycle. Food and water were available ad libitum. The ethanolic kava product was dried under vacuum to remove the solvent. The oily residue was dissolved in polyethylene glycol 400 (PEG400) at a concentration of 5 mg/mL. Mice (n = 3 per group) were administered kava or vehicle (200 μL) through the *per os* route and euthanized by CO_2_ administration at various time points post-administration. Urines were collected by following our reported procedure [42]. Briefly, each mouse was placed on a clean piece of aluminum foil and urine was passively released upon CO_2_ euthanasia. Blood (200–250 μL) was collected from mice by cardiac puncture with serum prepared. Lung, liver, and brain were harvested and flash frozen in liquid nitrogen. All samples were stored at– 80 °C until processed.

Each sample was processed individually with kavalactones recovered by an ethyl acetate extraction followed by a solid phase extraction [37]. Briefly, mouse liver, lung or brain tissues (~5 mg) were mechanically homogenized in H_2_O (180 *μ*L, LC-MS grade) with 0.1% formic acid (HCO_2_H). The [^2^H_2_]-DHM internal standard was added at the level of 50 pg/mg tissue. After sonication for 5 min, tissue homogenate (20 *μ*L) was mixed with methanol (900 *μ*L, -20 °C). Mouse serum or urine samples (5 *μ*L), added with [^2^H_2_]-DHM at the level of 100 pg/*μ*L, were mixed with cold CH_3_OH (495 *μ*L, -20 °C). From here, all samples were processed following the same procedures. After vortexing, the mixture was kept at -20 °C for 30 min, and centrifuged at 13,000 g for 20 min to remove the proteins and debris. The supernatant was vacuum centrifuged to dryness and resuspended in H_2_O (100 *μ*L). Kavalactones were extracted with ethyl acetate (600 *μ*L), vacuum centrifuged to dryness, resuspended in 10% CH_3_OH in H_2_O (1 mL), followed by a solid-phase extraction with a SOLA HRP cartridge (Thermo Fisher) (10 mg), pre-conditioned with CH_3_OH (1 mL) and H_2_O (1 mL). After wash with 10% CH_3_OH in H_2_O (2 mL), kavalactones were eluted with 100% CH_3_OH (1 mL). The elute was vacuum centrifuged to dryness, resuspended in 10% CH_3_OH in H_2_O with 0.1% HCO_2_H (100 *μ*L), and analyzed with the same method.

Peak plasma concentration (C_max_) of kavain, DHK, methysticin, DHM and desmethoxyyangonin and time to reach the C_max_ (t_max_) in serum/tissue homogenates were recorded directly from the raw data of concentration-time profile. The mean concentration-time data of serum (ng/mL) and tissue (liver, lung and brain) homogenates (ng/g) were subjected to non-compartmental analysis using Phoenix^™^, version 6.4.0.768 (Certara Inc, Missouri, USA). The area under the serum/tissue concentration-time up to the last observation (AUC_0-t_) was calculated using the linear trapezoidal method. Mean residence time (MRT) was calculated as AUMC_0-t_/AUC_0-t_ ratio, where AUMC_0-t_ is the area under the first moment curve up to the last observation. Oral clearance (Cl/F) was determined as Cl/F = Dose/AUC_0-t_.

### Human studies and kavalactone quantifications

The study was approved by the IRB at the University of Minnesota and all subjects provided informed, written consent. Urine and plasma samples were obtained from healthy adult smokers, who took the soft-gel kava three times daily for 7 days. A spot urine sample prior to kava and a 24-h urine sample on day 6–7 of the 7-day kava intervention were collected. Plasmas were obtained prior to kava and on days 6 or 7. All samples were stored at -80 °C until analyzed. Briefly, [^2^H_2_]-DHM was added as the internal standard (1 pg/*μ*L). The plasma samples (100 *μ*L) was mixed with CH_3_OH (1 mL, -20 °C) to precipitate proteins. The supernatant was vacuum centrifuged to dryness and resuspended in 10% CH_3_OH in H_2_O (100 *μ*L). Such plasma samples or urine samples were further processed via ethyl acetate and solid phase extraction as detailed for the mouse study, followed by UPLC-MS/MS analysis.

### Statistical analysis

Data were presented as mean ± standard deviation (SD). Differences were evaluated by two-tailed student *t*-test analysis at 95% confidence interval using SigmaPlot 12.0 (Systat Software Inc., San Jose, CA, USA).

## Results and discussions

### Mass spectrometric characterization of kavalactones and method validation

The observed ions [M+H]^+^ of the pure kavalactones at the full MS scan stage had excellent agreement with the calculated *m/z* values (within 1 ppm). Their reconstructed ion chromatograms and product ion spectra were also assayed by online UPLC-MS/MS (S1 Fig). Such product ion spectra had excellent agreement with the spectra acquired by direct infusion. Kavain, as an example, had major product ions at *m/z* 115.0541 (observed vs. calculated 115.0542, Δ0.9 ppm), *m/z* 153.0693 (observed vs. calculated 153.0699, Δ3.9 ppm) and *m/z* 185.0954 (observed vs. calculated 185.0961, Δ3.8 ppm).

The calibration curves of DHM using the ions at *m/z* 131.0491, 135.0441 and 161.0597 in seven different matrices were constructed (S2 Fig). Its concentration ranges were selected based on the amount of DHM detected in the analyzed samples. The linearity of all calibration curves is excellent. The LOD value is typically estimated as 3 times of the signal to noise ratio based upon the guidance recommended by ICH Q2(R1) [43]. However, there is no measurable background signal at the MS/MS scan stage in the blank sample acquired by the high resolution Orbitrap. Therefore, we estimated LOD and LOQ by 3.3*σ*/*s* and 10*σ*/*s* respectively (*σ* is the standard deviation of the slope (*s*) of the calibration curve) [43, 44]. The LOD and LOQ values of DHM in each matrix are summarized in S1 Table. Since kavain, DHK, methysticin and desmethoxyyangonin are structurally similar to DHM and we do not have isotope-labeled standards for each of them, [^2^H_2_]-DHM was used as the internal standard. Their LOD and LOQ were estimated (S1 Table) by factoring the ratios of the peak areas of individual kavalactones to the peak area of the same amount of [^2^H_2_]-DHM [45] and assuming that ionization efficiency of kavalactones at the concentration range of the calibration curves are the same.

The selectivity of the method was evaluated by measuring the kavalactones in the control samples with/without spiking kavalactones at the lower limit of detection (LLOD) level. Via the accurate measurements of the Orbitrap, we can selectively detect these five kavalactones in different matrices by extracting product ions at a 5 ppm mass tolerance window of the exact mass. There were no kavalactones detected in the control samples while all kavalactones were detected in the spiked samples with 6–10 scans across the full width of the peak (S3 Fig). Similarly, 10–14 scans were acquired across the full width of the peak at the level of LOQ, suitable for quantitative analysis by the high resolution Orbitrap MS/MS [36]. The accuracy, precision and reproducibility of the method in different matrices are summarized in Table 1 and S2–S7 Tables. Overall, in the matrices spiked with LLOD level of kavalactones, the method showed good accuracy (± 20%). Intraday and interday precision values are 3.9–18% and 4.1–18.3%, respectively. At spiking levels 3 times LLOD and above, the overall accuracy was excellent (± 15%). The intraday and interday precision values were 2.1–10.5% and 2.5–11.8%, respectively.

**Table 1:** Accuracy, and intraday and interday precision of DHM in the mouse liver tissues.

	Spiked DHM level (pg/mg tissue)	Day 1	Day 2	Day 3	CV (%) within-day ^a^	CV (%) between-day ^a^
Mean	5	4.8 (95.6%) ^b^	5.0 (100.5%) ^b^	4.8 (96.4%) ^b^	5.8	5.9
SD		0.3	0.2	0.3		
RSD		7.3	3.7	6.3		
Mean	15	14.1 (93.8%) ^b^	14.0 (93.2%) ^b^	12.2 (81.8%) ^b^	3.2	8.0
SD		0.5	0.5	0.3		
RSD		3.6	3.6	2.0		
Mean	50	53.2 (106.2%) ^b^	50.9 (101.8%) ^b^	46.6 (93.2%) ^b^	10.5	11.8
SD		6.5	5.3	3.6		
RSD		12.2	10.4	7.8		
Mean	4500	4455.0 (99.0%) ^b^	4396.5 (97.7%) ^b^	4545.0 (101.0%) ^b^	3.5	3.6
SD		118.4	110.3	216.0		
RSD		2.7	2.5	4.8		

Accuracy, and intraday and interday precision of DHM (pg/mg tissue) in the control mouse liver tissues with DHM spiking level of 5, 15, 50 and 4500 pg/mg tissue.

^a^ Within-day and between-day estimates were conducted with 6 independent measurements on three different days.

^b^ Values in parentheses represent accuracy of the method.

### The composition of five kavalactones in two commercial kava

For the ethanolic kava extract, DHK (0.247 ± 0.018 g/ g kava) and kavain (0.172 ± 0.030 g/ g kava) were the most abundant kavalactones, followed by desmethoxyyangonin (0.103 ± 0.011 g/ g kava), DHM (0.089 ± 0.008 g/ g kava), and methysticin (0.021 ± 0.009 g/ g kava) (S8 Table). These five kavalactones account for ~ 63% of the mass of the ethanolic kava extract. Their abundance was generally higher than that determined by us before via the large-scale isolation [38]. The lower value from isolation was at least partially due to the inevitable loss of materials during isolation. Overall, these data demonstrated that the five kavalactones were the major kavalactones in the ethanolic kava. For the soft-gel kava, the estimated total amount of these five major kavalactones was 77 ± 4 mg/capsule, consistent with the labeled 75 mg total kavalactones per capsule. However, the relative abundance of these five kavalactones was quite different from that in the ethanolic kava extract (S8 Table). The relative abundance of methysticin in the kava capsule was 3.1 times to that in the ethanolic kava extract. On the other hand, the abundance of DHK in the kava capsule was less than half of that in the ethanolic kava extract. The relative abundance of the other three kavalactones also differs significantly. These results substantiate the fact that dietary supplement kava products on the market can differ significantly in their chemical compositions, displaying different pharmacology and revealing different safety profiles.

### Pharmacokinetic studies of kava in C57BL/6J mice

**Fig 2:**
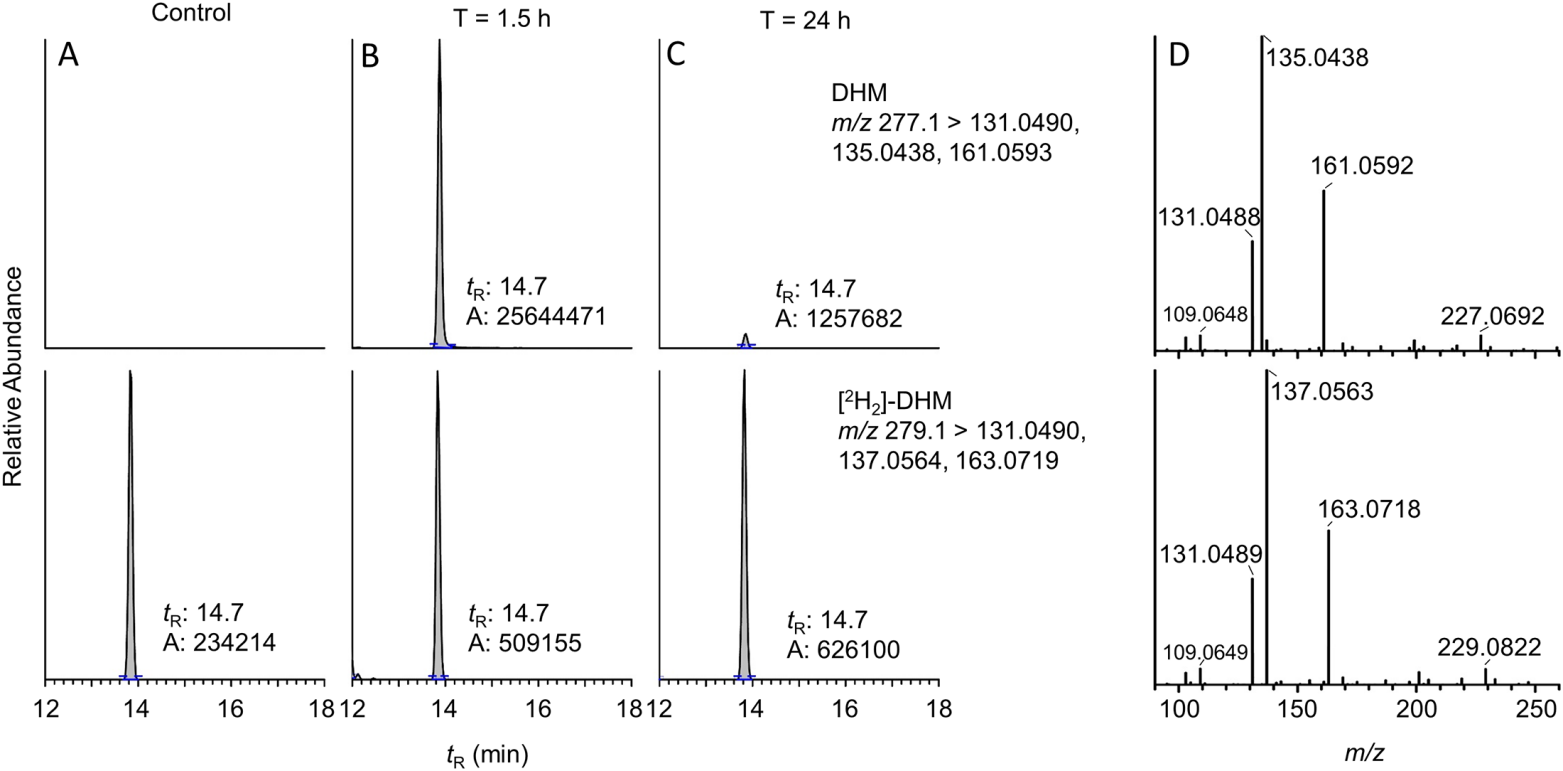
Targeted UPLC-MS/MS analysis of DHM in mouse liver tissues. Reconstructed ion chromatograms of DHM in liver tissues from mice with no kava (A), 1.5 h (B) and 24 h (C) after kava administration. (D) Mass spectra of DHM and [^2^H_2_]-DHM (the internal standard). The mass extraction window was of ± 5 ppm. The scale of the signal of panel C was normalized to the response of DHM in panel B.

The reproducibility of the method was evaluated by measuring these kavalactones in the mouse liver tissues collected 1.5-h after kava treatment. The CV% values for the intraday and interday precision for DHM were 6.8% and 8.7% (S9 Table). Similar results were obtained for the other four kavalactones (S9 Table). Representative reconstructed ion chromatograms of DHM from the mouse liver tissues showed the sensitivity and specificity of the method (Fig 2A–2C). The mass spectrum of these kavalactones also had an excellent agreement with the standards (Fig 2D and S1 Fig).

**Fig 3:**
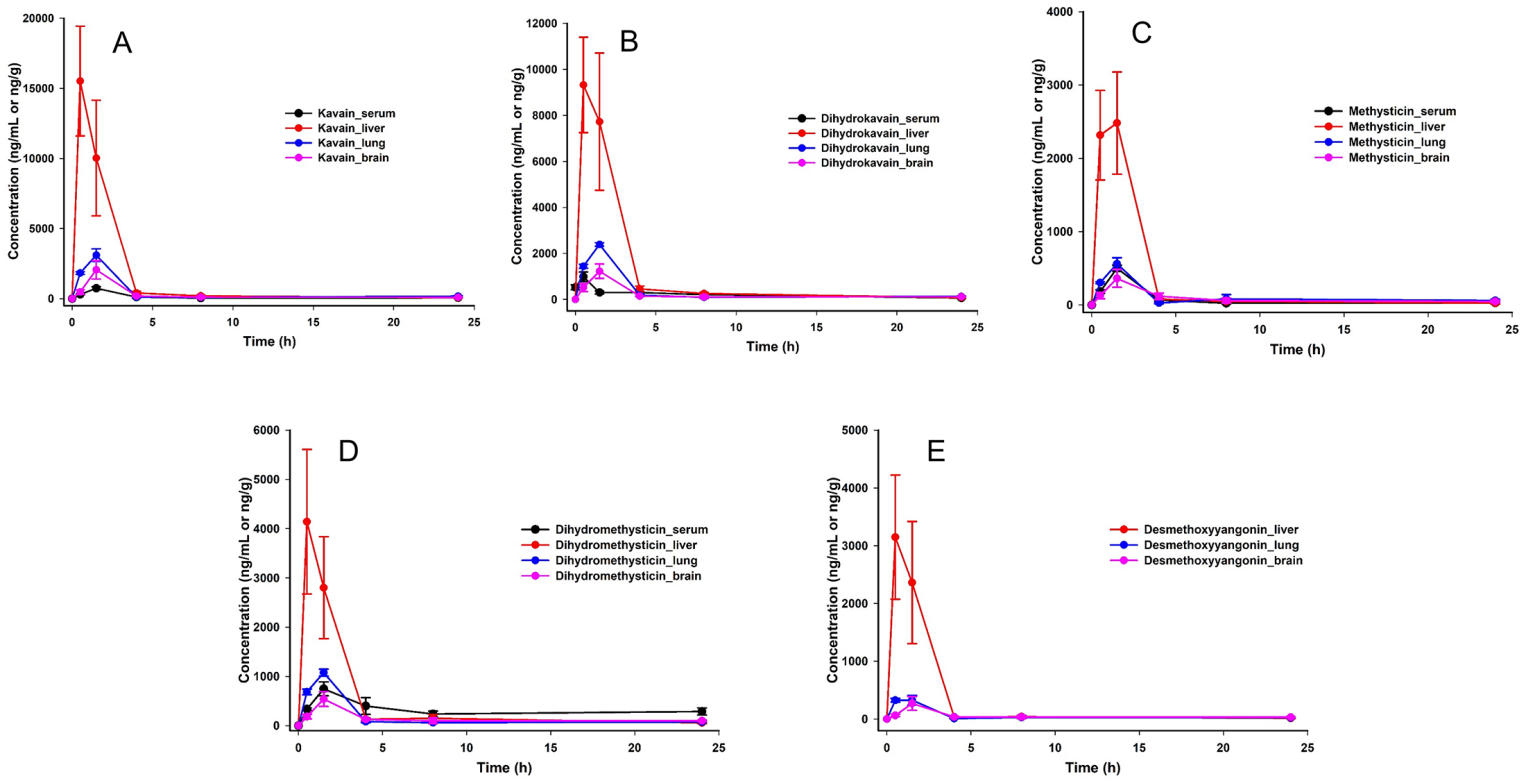
Pharmacokinetics and biodistribution of (A) kavain, (B) dihydrokavain, (C) methysticin, (D) dihydromethysticin, and (E) desmethoxyyangonin in the mouse serum, liver, lung, and brain. Samples were collected 0.5, 1.5, 4, 8, and 24 h after kava treatment. Mice with no kava treatment was used for 0-h timepoint.

Given that kavalactones account for around 60% of the mass of this kava extract (S8 Table), a single oral dose of kava at 41 mg/kg of bodyweight would be comparable to a dose of 150 mg total kavalactone for a human of 75 kg bodyweight according to the body surface area normalization method [46]. This is within the range of the recommended human daily dosage of kava [47]. As expected, kavalactones were below the LOD in all of the serum and tissue samples from the mice without kava treatment. In kava-treated mice, kavain, DHK, methysticin and DHM were above the LOQ in all samples (Fig 3). The amount of desmethoxyyangonin was less than the other kavalactones at the later-time point samples and was below the LOD in the serum samples at the 8- and 24-h time points. Interestingly, considerate amount of desmethoxyyangonin was detected in the earlier-time point urine samples (S4 Fig), suggesting that desmethoxyyangonin was quickly secreted. The highest concentrations of these kavalactones were achieved in liver tissues, reaching their maximum concentrations 0.5 h after kava oral administration. The concentrations of kavain and dihydrokavain in liver tissues could reach 10–15 μg/g tissues, equivalent to a concentration of 40–60 *μ*M. The highest abundance of the other kavalactones in the liver tissues were 2–4 μg/g tissues, equivalent to 10–15 *μ*M. In the lung and brain tissues, kavalactones were readily detected 0.5 h after kava administration as well. Their concentrations reached the maximum levels at the 1.5-h time point. The pharmacokinetics of these five kavalactones in the serum samples, however, were different from those in the tissues. Although the maximum abundance of these kavalactones were detected at the 1.5-h time point, there were relatively smaller dynamic changes of their abundance over the 24-h time period in comparison to the tissues, particularly for DHK and DHM that their concentrations remained at ~1 μM even 24 hours after the single dose kava exposure. The maximum concentrations of these kavalactones were between 2–4 *μ*M except for desmethoxyyangonin, which is below 0.5 *μ*M.

These data demonstrate that all five kavalactones are orally available and can cross the blood-brain barrier. They, kinetically, reached the liver tissues first, consistent with its oral route of administration [48]. These kavalactones, except desmethoxyyangonin, were in the low *μ*M concentrations in all tissues even 24 hours after the single oral dose of kava. Given their relatively slow clearance, a single-dose kava may result in long-term pharmacodynamics. Further investigation, therefore, is warranted to determine the optimal kava dosing frequency. The concentrations of these kavalactones in different tissues also provided information for future *ex vivo* experiments in the context of the *in vivo* relevance. Serum/tissue pharmacokinetic parameters of kavain, DHK, methysticin, DHM and desmethoxyyangonin are shown in S10 Table. Tissue-to-serum AUC_0-t_ ratios were highest for liver, suggesting maximum distribution of kavalactones in liver. They also showed adequate exposure to brain with brain-to-serum AUC_0-t_ ratio of 0.44 to 2.25. Among the studied kavalactones, kavain showed maximum affinity to brain with brain-to-serum AUC_0-t_ ratio of 2.25. The volume of distribution (V_d_/F, 11.0–41.6 L/h/kg) of kavain, dihydrokavain, methysticin and dihydromethysticin is larger than the total blood volume of mouse (0.085 L/kg)[49]. Moderate tissue-to-serum AUC_0-t_ ratio, large V_d_/F and long MRT (5.6–9.2 h) indicate the extensive affinity of kavalactones to the tissues. These five kavalactones also appeared to have differential tissue preference. Relatively more methysticin was retained in the brain with less in the urine (S4 Fig). On the other hand, the relative abundance of desmethoxyyangonin was higher in the urine than its natural abundance (S4 Fig), suggesting a quick clearance of desmethoxyyangonin *in vivo*.

### Quantification of five kavalactones in human plasma and urine samples

**Fig 4:**
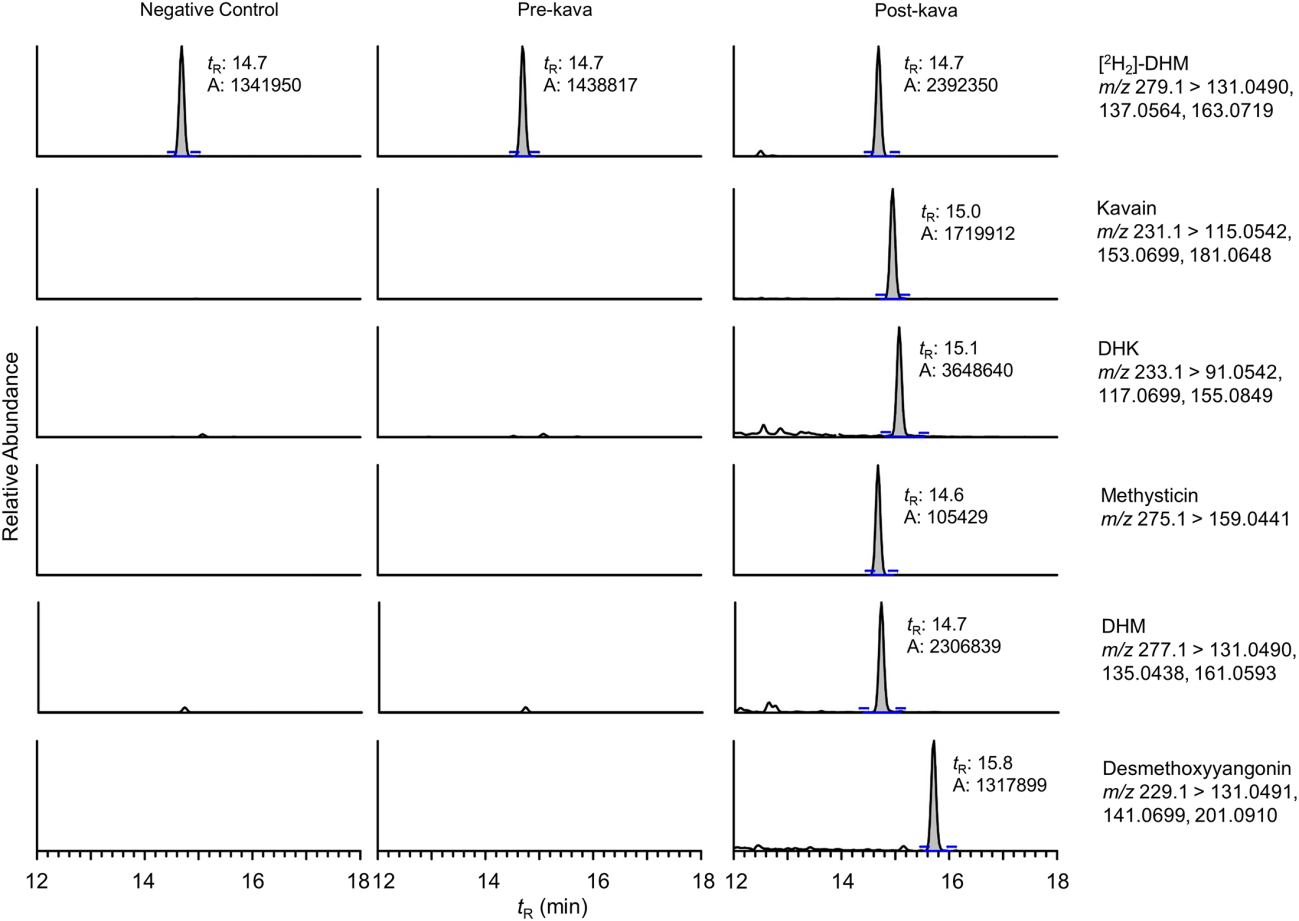
Reconstructed ion chromatograms at MS/MS scan stage of [^2^H_2_]-DHM, kavain, DHK, methysticin, DHM, and demethoxyyangonin from a control urine, and the urine samples collected from a subject pre- and post-kava. [^2^H_2_]-DHM was used as the internal standard. The mass extraction window was of ± 5 ppm.

None of the five kavalactones were above the LODs in the urine or plasma samples of the subjects before they started taking kava capsules (Fig 4 and Table 2), consistent with the fact that these kavalactones are unique to kava. Kavain, DHK, DHM and desmethoxyyangonin were above their LOQs in the post-kava urine samples while methysticin was below its LOD. Kavain, desmethoxyyangonin, and DHK were the most abundant kavalactones in the urine samples. All kavalactones were above their LOQs in the post-kava plasma samples. DHM was the most abundant kavalactone followed by DHK and kavain. Unlike in the urine samples, methysticin was well above the LOQ while desmethoxyyangonin was barely above the LOQ in both subjects. Consistent with the observation in C57BL/6 mice, these five kavalactones appeared to have different pharmacokinetics and biodistributions in humans (S5 Fig). The relative abundance of DHM/total kavalactones were considerably greater in the plasma than that in the urine while desmethoxyyangonin was dominantly detected in the urine samples. Methysticin, of comparable abundance as desmethoxyyangonin in the kava capsule, on the other hand, was readily detectable in the plasma but below its LOD in the urine samples. Kavain had a higher relative abundance in the urine relative to plasma while DHK had a higher abundance in the plasma relative to the urine.

**Table 2:** The amount of kavain, DHK, methysticin, DHM and desmethoxyyangonin in the urine and plasma samples of two subjects pre- and post-kava administration.

	Kavain	DHK	Methysticin	DHM	Desmethoxyyangonin
**Urine (pg/mL)**					
Subject 01 Pre-kava	ND	ND	ND	ND	ND
Subject 01 Post-kava	3019 ± 183 (43.4%)	1330 ± 144 (19.1%)	ND	984 ± 25 (14.1%)	1629 ± 181 (23.4%)
Subject 02 Pre-kava	ND	ND	ND	ND	ND
Subject 02 Post-kava	1775 ± 317 (30.7%)	1350 ± 96 (23.3%)	ND	688 ± 50 (11.9%)	1972 ± 281 (34.1%)
**Plasma (pg/mL)**					
Subject 01 Pre-kava	ND	ND	ND	ND	ND
Subject 01 Post-kava	9473 ± 86 (9.5%)	28765 ± 1252 (28.7%)	6959 ± 406 (6.9%)	54140 ± 3068 (54.1%)	808 ± 66 (0.8%)
Subject 02 Pre-kava	ND	ND	ND	ND	ND
Subject 02 Post-kava	18618 ± 827 (11.8%)	51037 ± 1308 (32.4%)	11732 ± 512 (7.5%)	75591 ± 652 (48.1%)	260 ± 90 (0.2%)

[^2^H_2_]-DHM was used as the internal standard (Mean ± SD, *n* = 2). The value in the parentheses is the percentage of individual kavalactone to total kavalactones. ND, below LOD.

## Conclusions

Given the increased popularity of kava in human usage [5], we developed and validated a sensitive UPLC-MS/MS method, employing the high-resolution accurate mass measurement by the Orbitrap at the MS/MS scan stage to quantify kavalactones in different matrices. By analyzing these five kavalactones in two kava products from the same company, our results demonstrated the chemical diversity of kava products and urged the need for better standardization to ensure its quality control and quality assurance in the future. This analytical method has also been used to characterize the pharmacokinetics the five kavalactones in C57BL/6J mice. The results demonstrate that the five kavalactones have distinct pharmacokinetics and biodistribution even though they are structurally similar. Interestingly, all kavalactones can cross the blood-brain barrier, supporting their potential for neurological effect. In addition, these kavalactones can be detected 24 hours after the single oral dose, raising the question how often kava needs to be administered. Lastly, this method was able to detect and quantify these five kavalactones in human plasma and urines after kava consumption based on the recommended regimen. Although not an exhaustive pharmacokinetic study, the current work clearly demonstrates the differing pharmacokinetics of these five kavalactones in humans. Systematic characterization of their pharmacokinetics in humans is warranted since these kavalactones may have different medical indications and influence the pharmacokinetics and pharmacodynamics of each other.

In summary, our method demonstrated a wider linear range for quantification (0.02–5 mg/g) and higher sensitivity (LOD: 27–155 pg/g) when employed to quantify kavalactones in kava products in comparison to previous methods (linear range: 0.25–1 mg/g; LOD: 0.5–1.1 *μ*g/mL) [31, 34, 35, 50, 51]. Similarly, previous pharmacokinetics study of kavain in rat needed 100 *μ*L plasma sample [14] while our method can quantify five kavalactones with 5 *μ*L serum or urine sample. This method was also able to quantify kavalactones from 100 *μ*L human urine and plasma samples, which has never been achieved before. These results demonstrate the scope of the UPLC-MS/MS method, which is critical to kava-related research and application.

## Supporting information

S1 Fig Reconstructed ion chromatograms of kavain, DHK, methysticin, DHM, and desmethoxyyangonin and their corresponding mass spectra. Equal amounts of individual standards (500 fg) were injected for UPLC-MS/MS analysis.
(TIF)

S2 Fig Calibration curve of DHM for (A) two kava products, (B) mouse liver, (C) mouse lung, (D) mouse brain, (E) mouse serum, (F) human urine and (G) human plasma using the product ions at *m/z* 131.0490, 137.0564 and 163.0719 at the MS/MS scan stage with a 5 ppm mass tolerance. (TIF)

S3 Fig Validation of selectivity. Reconstructed ion chromatograms of kavalactones in liver tissues of control mice without and with spiking kavalactones (3 pg/mg tissue). The mass extraction window was ± 5 ppm.
(TIF)

S4 Fig The relative abundance of (A) kavain, (B) DHK, (C) methysticin, (D) DHM and (E) desmethoxyyangonin in kava products and in mouse samples at different time points. (TIF)

S5 Fig The relative abundance of (A) kavain, (B) DHK, (C) methysticin, (D) DHM and (E) desmethoxyyangonin in the kava capsule, human urine and plasma. (TIF)

S1 Table LOD and LOQ values of kavalactones in different matrices. LOD and LOQ were estimated by the 3.3*σ*/*s* and 10*σ*/*s*, respectively (*σ* is the standard deviation of the slope (*s*) of the calibration curve).
(DOCX)

S2 Table Accuracy, and intraday and interday precision of kavain, DHK, methysticin and desmethoxyyangonin (pg/mg tissue) in the control mouse liver tissues at spiking level of 5, 15, 50 and 4500 pg /mg tissue. Within-day and between-day estimates were conducted with 6 independent measurements on three different days. Values in parentheses represent accuracy of the method.
(DOCX)

S3 Table Accuracy, and intraday and interday precision of kavain, DHK, methysticin, DHM and desmethoxyyangonin (pg/mg tissue) in the control mouse lung tissues at spiking level of 5, 15, 50 and 4500 pg/mg tissue. Within-day and between-day estimates were conducted with 6 independent measurements on three different days. Values in parentheses represent accuracy of the method.
(DOCX)

S4 Table Accuracy, and intraday and interday precision of kavain, DHK, methysticin, DHM and desmethoxyyangonin (pg/mg tissue) in the control mouse brain tissues at spiking level of 5, 15, 50 and 4500 pg/mg tissue. Within-day and between-day estimates were conducted with 6 independent measurements on three different days. Values in parentheses represent accuracy of the method.
(DOCX)

S5 Table Accuracy, and intraday and interday precision of kavain, DHK, methysticin, DHM and desmethoxyyangonin (pg/*μ*L) in the control mouse serum at spiking level of 0.8, 8, 80 and 8000 pg/μL. Within-day and between-day estimates were conducted with 6 independent measurements on three different days. Values in parentheses represent accuracy of the method.
(DOCX)

S6 Table Accuracy, and intraday and interday precision of kavain, DHK, methysticin, DHM and desmethoxyyangonin (pg/*μ*L) in the plasma of pre-kava human subjects at spiking level of 0.15, 0.4, 1 and 2 pg/*μ*L. Within-day and between-day estimates were conducted with 6 independent measurements on three different days. Values in parentheses represent accuracy of the method.
(DOCX)

S7 Table Accuracy, and intraday and interday precision of kavain, DHK, methysticin, DHM and desmethoxyyangonin (pg/*μ*L) in the urine of pre-kava human subjects at spiking level of 0.15, 0.4, 1 and 2 pg/*μ*L. Within-day and between-day estimates were conducted with 6 independent measurements on three different days. Values in parentheses represent accuracy of the method.
(DOCX)

S8 Table UPLC-MS/MS analysis of the composition of two kava products (Mean ± SD, n = 3). The value in parentheses is the relative amount of individual kavalactone to the total kavalactones.
(DOCX)

S9 Table Within-day and between-day estimates of kavain, DHK, methysticin, DHM and desmethoxyyangonin (pg/mg tissue) in the 1.5-h mouse liver tissues. Within-day and between-day estimates were conducted with three independent measurements on three different days.
(DOCX)

S10 Table Pharmacokinetic parameters of kavain, DHK, methysticin, DHM and desmethoxyyangonin. (DOCX)
