# Supplementary figures and images for: A stable isotope dilution tandem mass spectrometry method of major kavalactones and its applications
Source: PLoS One. 2018 May 24;13(5):e0197940. doi: 10.1371/journal.pone.0197940 (PMC5993114; doi:10.1371/journal.pone.0197940)

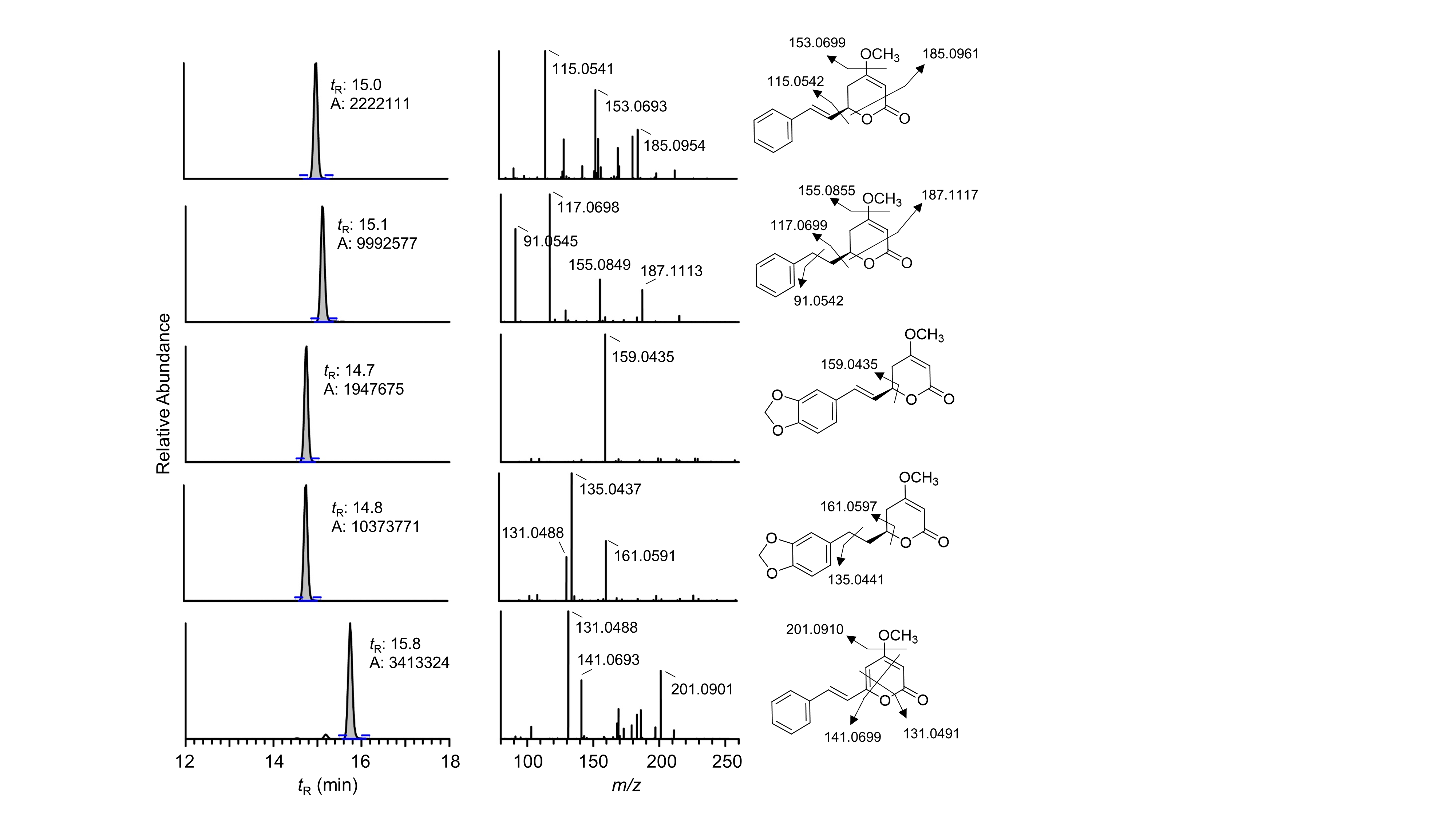

Supplement: S1 Fig — Equal amounts of individual standards (500 fg) were injected for UPLC-MS/MS analysis. (TIF) [file pone.0197940.s001.tif]

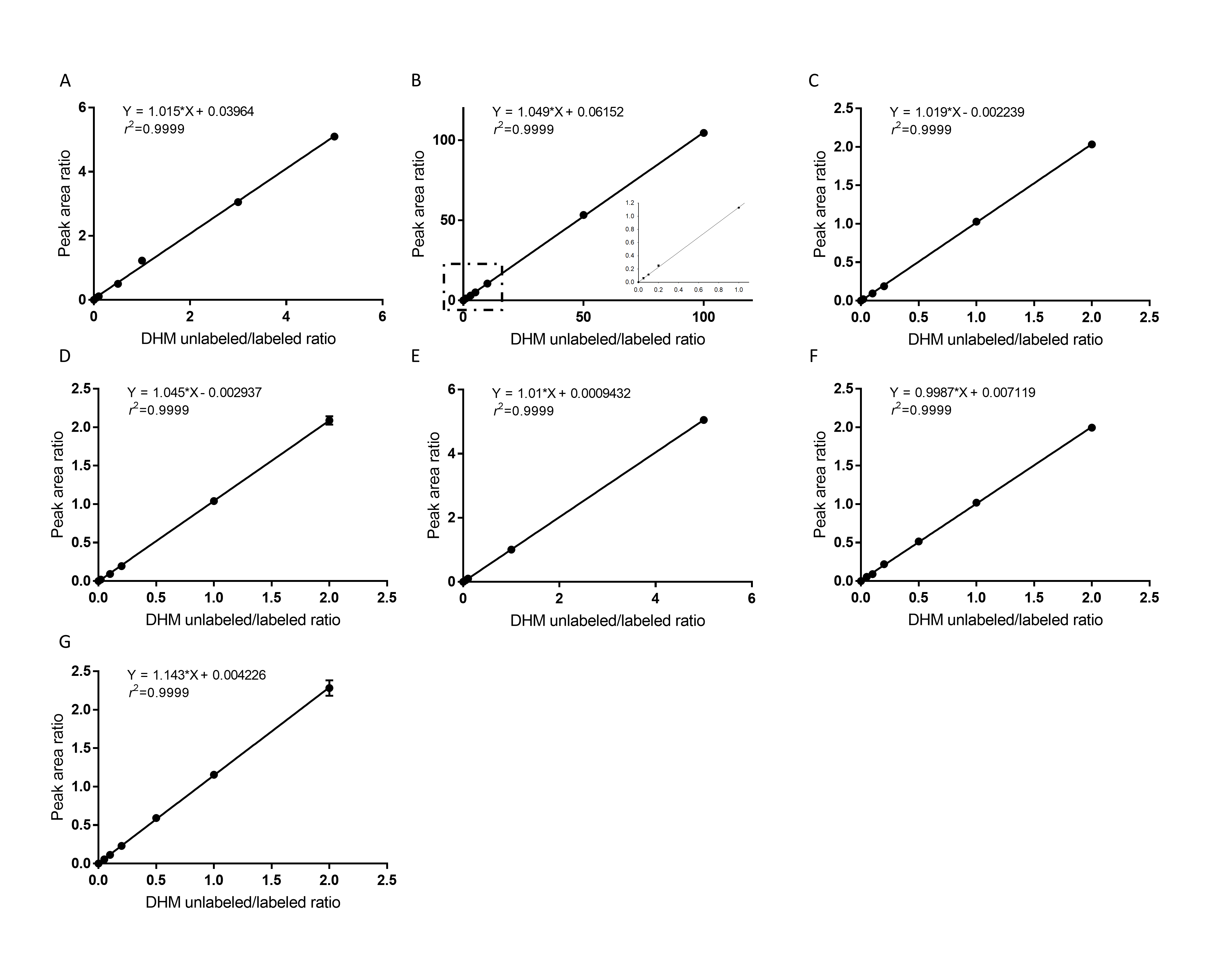

Supplement: S2 Fig — (TIF) [file pone.0197940.s002.tif]

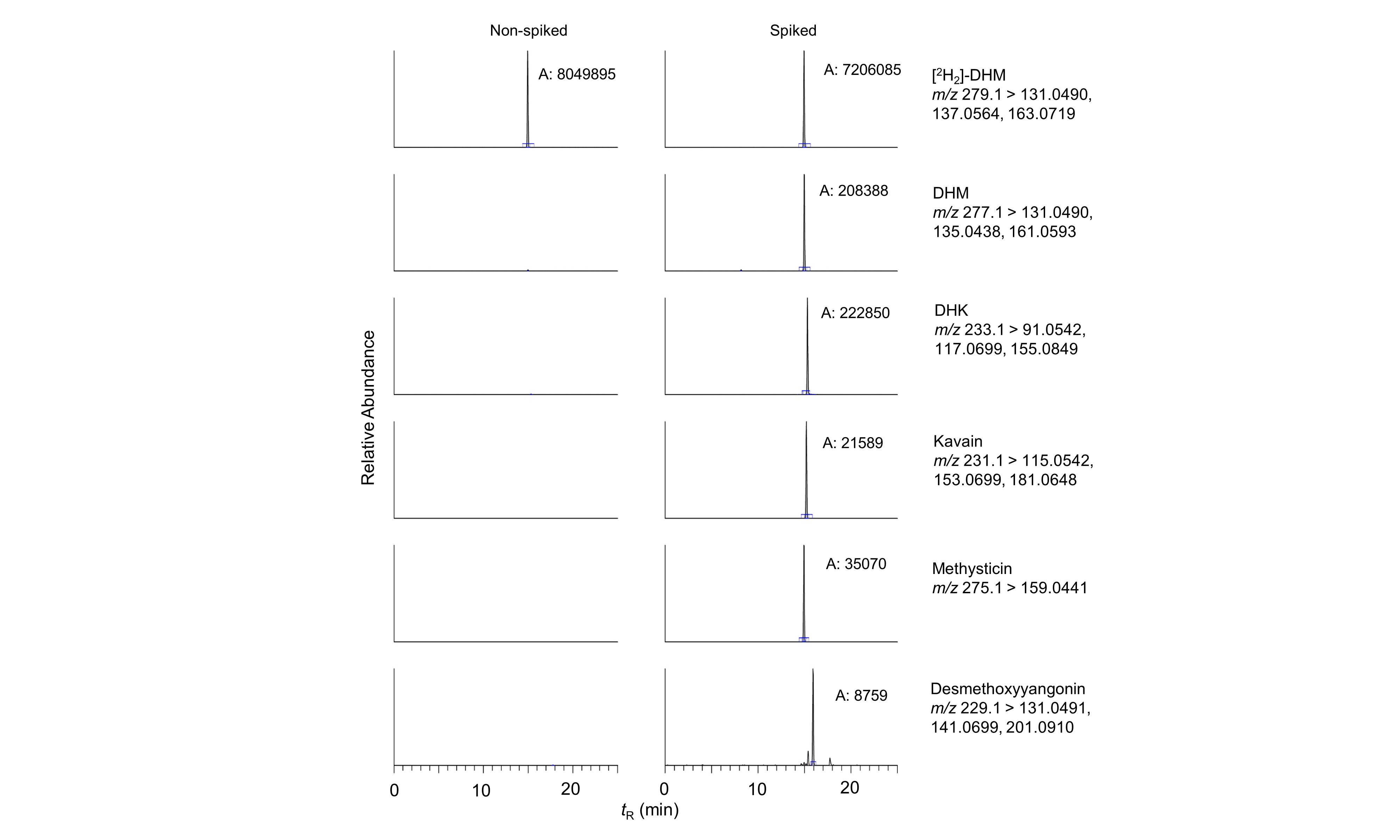

Supplement: S3 Fig — Reconstructed ion chromatograms of kavalactones in liver tissues of control mice without and with spiking kavalactones (3 pg/mg tissue). The mass extraction window was ± 5 ppm. (TIF) [file pone.0197940.s003.tif]

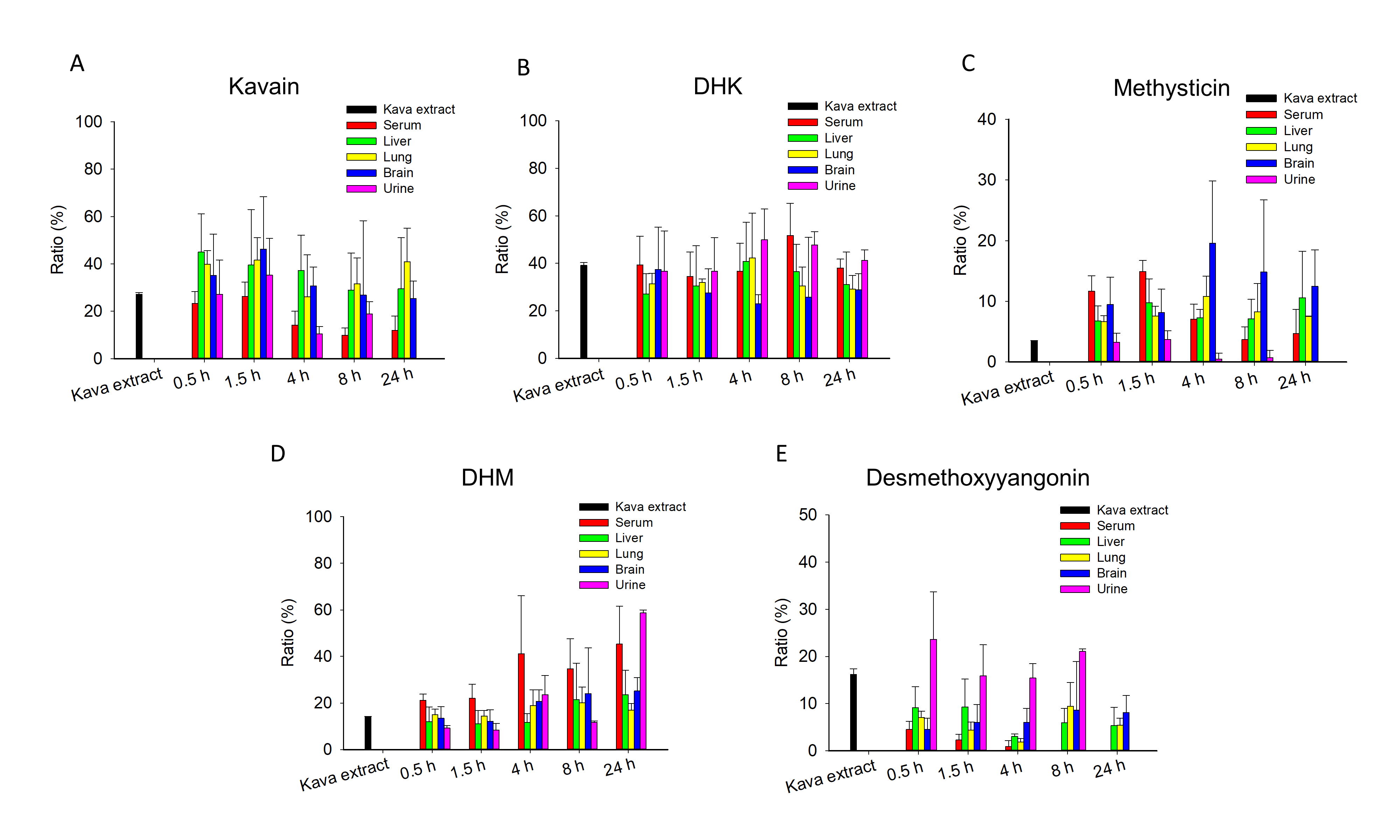

Supplement: S4 Fig — (TIF) [file pone.0197940.s004.tif]

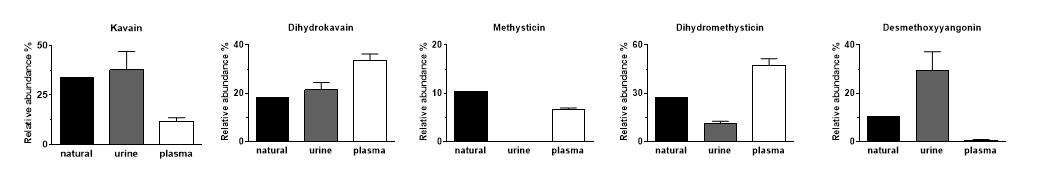

Supplement: S5 Fig — (TIF) [file pone.0197940.s005.tif]
