# Supplementary material for: A stable isotope dilution tandem mass spectrometry method of major kavalactones and its applications
Source: PLoS One. 2018 May 24;13(5):e0197940. doi: 10.1371/journal.pone.0197940 (PMC5993114; doi:10.1371/journal.pone.0197940)
Supplement: S1 Table — LOD and LOQ were estimated by the 3.3σ/s and 10σ/s, respectively (σ is the standard deviation of the slope (s) of the calibration curve). (DOCX) [file pone.0197940.s006.docx]

**S1 Table. LOD and LOQ values of kavalactones in different matrices.**

|  | **Kavain** | **DHK** | **Methysticin** | **DHM** | **Desmethoxyyangonin** |
| --- | --- | --- | --- | --- | --- |
| **Kava pill (pg/mL)** | | | | | |
| LOD | 118 | 26 | 155 | 27 | 81 |
| LOQ | 353 | 77 | 464 | 81 | 243 |
| **Mouse liver (pg/mg tissue)** | | | | | |
| LOD | 3.3 | 0.7 | 4.3 | 0.8 | 2.3 |
| LOQ | 10.0 | 2.1 | 13.1 | 3.0 | 6.9 |
| **Mouse lung (pg/mg tissue)** | | | | | |
| LOD | 2.6 | 0.6 | 3.4 | 0.6 | 1.8 |
| LOQ | 7.4 | 1.6 | 9.7 | 1.7 | 5.1 |
| **Mouse brain (pg/mg tissue)** | | | | | |
| LOD | 2.2 | 0.5 | 2.9 | 0.5 | 1.5 |
| LOQ | 6.1 | 1.3 | 8.0 | 1.4 | 4.2 |
| **Mouse serum (pg/mL)** | | | | | |
| LOD | 817.8 | 178.4 | 1072.5 | 187.4 | 562.2 |
| LOQ | 2477.9 | 540.5 | 3249.5 | 567.8 | 1703.4 |
| **Human urine (pg/mL)** | | | | | |
| LOD | 105.8 | 18.6 | 137.0 | 24.8 | 73.0 |
| LOQ | 319.7 | 55.7 | 416.5 | 71.8 | 221.2 |
| **Human plasma (pg/mL)** | | | | | |
| LOD | 68.9 | 15.0 | 90.4 | 15.8 | 47.4 |
| LOQ | 209.5 | 45.7 | 274.7 | 48.0 | 144.0 |

LOD and LOQ were estimated by the 3.3*σ*/*s* and 10*σ*/*s,* respectively (*σ* is the standard deviation of the slope (*s*) of the calibration curve)
