# Supplementary material for: A stable isotope dilution tandem mass spectrometry method of major kavalactones and its applications
Source: PLoS One. 2018 May 24;13(5):e0197940. doi: 10.1371/journal.pone.0197940 (PMC5993114; doi:10.1371/journal.pone.0197940)
Supplement: S2 Table — Within-day and between-day estimates were conducted with 6 independent measurements on three different days. Values in parentheses represent accuracy of the method. (DOCX) [file pone.0197940.s007.docx]

**S2 Table. Accuracy, and intraday and interday precision of kavain, DHK, methysticin and desmethoxyyangonin (pg/mg tissue) in the control mouse liver tissues at spiking level of 5, 15, 50 and 4500 pg /mg tissue.**

|  | **Spiked level (pg/mg tissue)** | **Day 1** | **Day 2** | **Day 3** | **Within-day (CV%)** | **Between-day (CV%)** |
| --- | --- | --- | --- | --- | --- | --- |
| **Kavain** | | | | | | |
| Mean | 5.0 | 4.7 (93.9%) | 5.3 (105.9%) | 4.7 (93.0%) | 14.4 | 15.2 |
| SD |  | 1.0 | 0.5 | 0.4 |  |  |
| RSD |  | 21.4 | 9.2 | 8.7 |  |  |
| Mean | 15.0 | 17.4 (115.9%) | 17.6 (117.4%) | 17.9 (119.4%) | 6.2 | 5.9 |
| SD |  | 1.1 | 1.5 | 0.6 |  |  |
| RSD |  | 6.3 | 8.3 | 3.6 |  |  |
| Mean | 50.0 | 48.7 (97.4%) | 46.5 (93.0%) | 43.6 (87.2%) | 5.3 | 7.3 |
| SD |  | 1.5 | 1.1 | 3.9 |  |  |
| RSD |  | 3.0 | 2.4 | 9.0 |  |  |
| Mean | 4500.0 | 4554.0 (101.2%) | 4536.0 (100.8%) | 4675.5 (103.9%) | 2.1 | 2.5 |
| SD |  | 134.4 | 63.4 | 71.7 |  |  |
| RSD |  | 3.0 | 1.4 | 1.5 |  |  |
| **DHK** | | | | | | |
| Mean | 5.0 | 5.2 (103.1%) | 5.3 (105.7%) | 5.3 (106.5%) | 5.8 | 5.5 |
| SD |  | 0.4 | 0.3 | 0.1 |  |  |
| RSD |  | 7.6 | 6.0 | 2.8 |  |  |
| Mean | 15.0 | 17.1 (113.9%) | 17.8 (118.9%) | 16.4 (109.4%) | 7.8 | 8.2 |
| SD |  | 1.5 | 0.4 | 1.8 |  |  |
| RSD |  | 2.5 | 8.3 | 10.7 |  |  |
| Mean | 50.0 | 46.8 (93.6%) | 50.9 (101.8%) | 49.1 (98.2%) | 10.2 | 10.2 |
| SD |  | 1.7 | 5.0 | 6.9 |  |  |
| RSD |  | 3.6 | 9.9 | 14.1 |  |  |
| Mean | 4500.0 | 4410.0 (98.0%) | 4554.0 (101.2%) | 4441.5 (98.7%) | 8.3 | 7.8 |
| SD |  | 607.5 | 153.8 | 182.2 |  |  |
| RSD |  | 13.7 | 3.4 | 4.1 |  |  |
| **Methysticin** | | | | | | |
| Mean | 5.0 | 4.6 (92.3%) | 4.7 (94.0%) | 4.1 (82.1%) | 11.1 | 12.5 |
| SD |  | 0.7 | 0.4 | 0.2 |  |  |
| RSD |  | 15.7 | 7.9 | 4.9 |  |  |
| Mean | 15.0 | 13.2 (88.8%) | 13.0 (86.8%) | 13.0 (86.6%) | 3.2 | 3.3 |
| SD |  | 0.2 | 0.3 | 0.6 |  |  |
| RSD |  | 1.5 | 2.5 | 4.9 |  |  |
| Mean | 50.0 | 52.3 (104.6%) | 49.8 (99.6%) | 49.7 (99.4%) | 4.9 | 5.3 |
| SD |  | 3.4 | 0.9 | 2.4 |  |  |
| RSD |  | 6.5 | 1.9 | 4.9 |  |  |
| Mean | 4500.0 | 4590.0 (102.0%) | 4549.5 (101.1%) | 4716.0 (104.8%) | 2.6 | 3.0 |
| SD |  | 181.7 | 60.0 | 76.3 |  |  |
| RSD |  | 3.9 | 1.3 | 1.6 |  |  |
| **Desmethoxyyangonin** | | | | | | |
| Mean | 5.0 | 4.9 (98.9%) | 5.1 (102.7%) | 4.5 (89.9%) | 17.4 | 17.5 |
| SD |  | 1.1 | 0.8 | 0.1 |  |  |
| RSD |  | 22.3 | 14.8 | 1.7 |  |  |
| Mean | 15.0 | 12.7 (84.8%) | 14.5 (96.6%) | 13.8 (92.1%) | 7.0 | 9.2 |
| SD |  | 0.9 | 0.9 | 1.0 |  |  |
| RSD |  | 7.2 | 6.2 | 7.6 |  |  |
| Mean | 50.0 | 48.5 (97.0%) | 44.1 (88.2%) | 45.0 (90.0%) | 3.8 | 6.3 |
| SD |  | 1.2 | 0.2 | 2.7 |  |  |
| RSD |  | 2.4 | 0.4 | 6.0 |  |  |
| Mean | 4500.0 | 4446.0 (98.8%) | 4423.5 (98.3%) | 4666.5 (103.7%) | 2.7 | 3.9 |
| SD |  | 133.3 | 102.3 | 134.4 |  |  |
| RSD |  | 3.0 | 2.3 | 2.9 |  |  |

Within-day and between-day estimates were conducted with 6 independent measurements on three diﬀerent days. Values in parentheses represent accuracy of the method
