# Supplementary material for: A stable isotope dilution tandem mass spectrometry method of major kavalactones and its applications
Source: PLoS One. 2018 May 24;13(5):e0197940. doi: 10.1371/journal.pone.0197940 (PMC5993114; doi:10.1371/journal.pone.0197940)
Supplement: S3 Table — Within-day and between-day estimates were conducted with 6 independent measurements on three different days. Values in parentheses represent accuracy of the method. (DOCX) [file pone.0197940.s008.docx]

**S3 Table. Accuracy, and intraday and interday precision of kavain, DHK, methysticin, DHM and desmethoxyyangonin (pg/mg tissue) in the control mouse lung tissues at spiking level of 5, 15, 50 and 4500 pg/mg tissue.**

|  | **Spiked level (pg/mg tissue)** | **Day 1** | **Day 2** | **Day 3** | **Within-day (CV%)** | **Between-day (CV%)** |
| --- | --- | --- | --- | --- | --- | --- |
| **Kavain** | | | | | | |
| Mean | 5.0 | 4.3 (85.6%) | 4.8 (95.9%) | 4.9 (97.1%) | 14.9 | 15.1 |
| SD |  | 0.9 | 0.5 | 0.7 |  |  |
| RSD |  | 21.2 | 9.9 | 14.4 |  |  |
| Mean | 15.0 | 14.9 (99.0%) | 15.3 (102.0%) | 14.7 (98.1%) | 3.5 | 3.8 |
| SD |  | 0.7 | 0.4 | 0.3 |  |  |
| RSD |  | 5.0 | 2.7 | 2.2 |  |  |
| Mean | 50.0 | 51.4 (102.7%) | 52.1 (104.2%) | 52.5 (105.0%) | 3.8 | 3.6 |
| SD |  | 2.9 | 0.7 | 1.7 |  |  |
| RSD |  | 5.6 | 1.4 | 3.2 |  |  |
| Mean | 4500.0 | 4428.6 (98.4%) | 4390.3 (97.6%) | 4622.0 (102.7%) | 4.3 | 4.8 |
| SD |  | 209.8 | 188.0 | 172.7 |  |  |
| RSD |  | 4.7 | 4.3 | 3.7 |  |  |
| **DHK** | | | | | | |
| Mean | 5.0 | 4.7 (93.6%) | 5.7 (114.1%) | 4.5 (90.8%) | 10.3 | 16.0 |
| SD |  | 0.5 | 0.3 | 0.6 |  |  |
| RSD |  | 11.1 | 5.7 | 14.0 |  |  |
| Mean | 15.0 | 15.7 (104.6%) | 14.6 (97.3%) | 14.1 (94.1%) | 7.6 | 8.8 |
| SD |  | 1.2 | 1.0 | 1.3 |  |  |
| RSD |  | 7.4 | 6.7 | 9.1 |  |  |
| Mean | 50.0 | 47.4 (94.7%) | 49.9 (99.9%) | 57.5 (114.9%) | 5.9 | 11.8 |
| SD |  | 4.2 | 1.8 | 2.3 |  |  |
| RSD |  | 9.0 | 3.6 | 4.0 |  |  |
| Mean | 4500.0 | 5085.4 (113.0%) | 4374.1 (97.2%) | 4551.8 (101.2%) | 4.8 | 9.3 |
| SD |  | 256.4 | 111.4 | 247.3 |  |  |
| RSD |  | 5.0 | 2.5 | 5.4 |  |  |
| **Methysticin** | | | | | | |
| Mean | 5.0 | 4.6 (91.3%) | 4.8 (96.0%) | 4.5 (90.8%) | 4.2 | 4.9 |
| SD |  | 0.2 | 0.2 | 0.2 |  |  |
| RSD |  | 5.2 | 3.4 | 3.8 |  |  |
| Mean | 15.0 | 15.3 (102.1%) | 15.1 (100.3%) | 15.6 (104.0%) | 5.4 | 5.3 |
| SD |  | 0.8 | 1.0 | 0.5 |  |  |
| RSD |  | 5.4 | 6.8 | 3.4 |  |  |
| Mean | 50.0 | 51.2 (102.3%) | 51.0 (102.0%) | 52.2 (104.3%) | 3.1 | 3.1 |
| SD |  | 2.1 | 1.0 | 1.5 |  |  |
| RSD |  | 4.1 | 1.9 | 2.9 |  |  |
| Mean | 4500.0 | 4466.2 (99.2%) | 4422.0 (98.3%) | 4691.4 (104.3%) | 3.3 | 4.3 |
| SD |  | 150.7 | 157.6 | 134.6 |  |  |
| RSD |  | 3.4 | 3.6 | 2.9 |  |  |
| **DHM** | | | | | | |
| Mean | 5.0 | 4.6 (92.7%) | 4.8 (96.7%) | 4.9 (98.8%) | 6.5 | 6.8 |
| SD |  | 0.2 | 0.5 | 0.2 |  |  |
| RSD |  | 3.5 | 9.7 | 4.3 |  |  |
| Mean | 15.0 | 14.9 (99.4%) | 14.6 (97.1%) | 14.4 (95.8%) | 3.1 | 3.4 |
| SD |  | 0.3 | 0.6 | 0.4 |  |  |
| RSD |  | 2.3 | 3.8 | 3.0 |  |  |
| Mean | 50.0 | 49.8 (99.6%) | 51.4 (102.8%) | 51.8 (103.6%) | 3.2 | 3.5 |
| SD |  | 1.4 | 1.0 | 2.2 |  |  |
| RSD |  | 2.9 | 1.9 | 4.2 |  |  |
| Mean | 4500.0 | 4416.6 (98.1%) | 4322.5 (96.1%) | 4545.2 (101.0%) | 3.5 | 4.1 |
| SD |  | 179.8 | 136.0 | 154.4 |  |  |
| RSD |  | 4.1 | 3.1 | 3.4 |  |  |
| **Desmethoxyyangonin** | | | | | | |
| Mean | 5.0 | 4.4 (89.0%) | 4.4 (88.7%) | 4.5 (90.1%) | 4.2 | 3.9 |
| SD |  | 0.2 | 0.1 | 0.3 |  |  |
| RSD |  | 3.5 | 2.8 | 5.7 |  |  |
| Mean | 15.0 | 15.4 (102.9%) | 16.9 (112.5%) | 14.7 (97.8%) | 5.0 | 8.5 |
| SD |  | 0.9 | 0.6 | 0.8 |  |  |
| RSD |  | 5.6 | 3.6 | 5.7 |  |  |
| Mean | 50.0 | 52.6 (105.3%) | 51.9 (103.7%) | 51.6 (103.2%) | 3.4 | 3.3 |
| SD |  | 2.2 | 1.6 | 1.2 |  |  |
| RSD |  | 4.2 | 3.2 | 2.4 |  |  |
| Mean | 4500.0 | 5109.1 (113.5%) | 4222.8 (93.8%) | 4692.9 (104.3%) | 3.4 | 10 |
| SD |  | 214.6 | 103.2 | 2.7 |  |  |
| RSD |  | 4.2 | 2.4 | 2.9 |  |  |

Within-day and between-day estimates were conducted with 6 independent measurements on three diﬀerent days. Values in parentheses represent accuracy of the method
