# Supplementary material for: A stable isotope dilution tandem mass spectrometry method of major kavalactones and its applications
Source: PLoS One. 2018 May 24;13(5):e0197940. doi: 10.1371/journal.pone.0197940 (PMC5993114; doi:10.1371/journal.pone.0197940)
Supplement: S4 Table — Within-day and between-day estimates were conducted with 6 independent measurements on three different days. Values in parentheses represent accuracy of the method. (DOCX) [file pone.0197940.s009.docx]

**S4 Table. Accuracy, and intraday and interday precision of kavain, DHK, methysticin, DHM and desmethoxyyangonin (pg/mg tissue) in the control mouse brain tissues at spiking level of 5, 15, 50 and 4500 pg/mg tissue.**

|  | **Spiked level (pg/mg tissue)** | **Day 1** | **Day 2** | **Day 3** | **Within-day (CV%)** | **Between-day (CV%)** |
| --- | --- | --- | --- | --- | --- | --- |
| **Kavain** | | | | | | |
| Mean | 5.0 | 4.7 (93.3%) | 5.2 (104.3%) | 4.6 (92.0%) | 11.2 | 12.5 |
| SD |  | 0.6 | 0.6 | 0.4 |  |  |
| RSD |  | 12.3 | 10.7 | 9.6 |  |  |
| Mean | 15.0 | 17.0 (113.6%) | 17.7 (118.0%) | 17.9 (119.1%) | 6.7 | 6.6 |
| SD |  | 0.9 | 1.4 | 1.1 |  |  |
| RSD |  | 5.3 | 8.2 | 6.0 |  |  |
| Mean | 50.0 | 51.8 (103.7%) | 51.8 (103.6%) | 52.4 (104.8%) | 4.1 | 3.8 |
| SD |  | 2.8 | 2.1 | 1.3 |  |  |
| RSD |  | 5.4 | 4.0 | 2.5 |  |  |
| Mean | 4500.0 | 4501.5 (100.0%) | 4445.4 (98.8%) | 4562.4 (101.4%) | 2.6 | 2.7 |
| SD |  | 141.1 | 130.0 | 59.5 |  |  |
| RSD |  | 3.1 | 2.9 | 1.3 |  |  |
| **DHK** | | | | | | |
| Mean | 5.0 | 4.5 (89.1%) | 5.9 (99.0%) | 4.6 (92.8%) | 5.6 | 7.4 |
| SD |  | 0.3 | 0.3 | 0.2 |  |  |
| RSD |  | 7.2 | 5.8 | 3.6 |  |  |
| Mean | 15.0 | 14.6 (97.0%) | 14.2 (94.4%) | 14.8 (98.7%) | 5.1 | 5.2 |
| SD |  | 0.2 | 1.2 | 0.4 |  |  |
| RSD |  | 1.7 | 8.4 | 2.9 |  |  |
| Mean | 50.0 | 53.1 (106.2%) | 47.1 (94.1%) | 56.3 (112.7%) | 8.2 | 11.6 |
| SD |  | 7.1 | 2.0 | 1.5 |  |  |
| RSD |  | 13.5 | 4.2 | 2.7 |  |  |
| Mean | 4500.0 | 4894.3 (108.8%) | 4515.3 (100.3%) | 4401.8 (97.8%) | 4.9 | 7.2 |
| SD |  | 360.5 | 112.7 | 94.9 |  |  |
| RSD |  | 7.4 | 2.5 | 2.2 |  |  |
| **Methysticin** | | | | | | |
| Mean | 5.0 | 5.6 (111.3%) | 4.4 (88.5%) | 4.5 (91.9%) | 14.1 | 18.3 |
| SD |  | 0.7 | 0.9 | 0.3 |  |  |
| RSD |  | 11.9 | 20.6 | 5.5 |  |  |
| Mean | 15.0 | 13.9 (92.7%) | 13.1 (87.0%) | 13.0 (86.3%) | 4.6 | 5.7 |
| SD |  | 0.1 | 1.0 | 0.4 |  |  |
| RSD |  | 1.0 | 7.5 | 3.1 |  |  |
| Mean | 50.0 | 52.5 (105.0%) | 50.3 (100.6%) | 52.7 (105.5%) | 3.5 | 4.1 |
| SD |  | 2.5 | 1.5 | 1.2 |  |  |
| RSD |  | 4.8 | 3.1 | 2.3 |  |  |
| Mean | 4500.0 | 4570.8 (101.6%) | 4532.8 (100.7%) | 4615.6 (102.6%) | 2.6 | 2.5 |
| SD |  | 99.4 | 112.4 | 140.1 |  |  |
| RSD |  | 2.2 | 2.5 | 3.0 |  |  |
| **DHM** | | | | | | |
| Mean | 5.0 | 5.5 (110.9%) | 4.5 (90.6%) | 4.4 (86.9%) | 6.7 | 15.3 |
| SD |  | 0.4 | 0.3 | 0.2 |  |  |
| RSD |  | 7.4 | 6.0 | 5.1 |  |  |
| Mean | 15.0 | 15.8 (105.2%) | 14.9 (99.3%) | 15.7 (104.9%) | 4.1 | 4.9 |
| SD |  | 0.5 | 0.8 | 0.6 |  |  |
| RSD |  | 3.3 | 5.2 | 3.9 |  |  |
| Mean | 50.0 | 50.4 (100.9%) | 49.7 (99.3%) | 50.8 (101.7%) | 3.4 | 3.3 |
| SD |  | 1.9 | 1.6 | 1.6 |  |  |
| RSD |  | 3.8 | 3.2 | 3.1 |  |  |
| Mean | 4500.0 | 4496.4 (99.9%) | 4417.9 (98.2%) | 4487.0 (99.7%) | 3.9 | 3.7 |
| SD |  | 159.2 | 150.3 | 203.1 |  |  |
| RSD |  | 3.5 | 3.4 | 4.5 |  |  |
| **Desmethoxyyangonin** | | | | | | |
| Mean | 5.0 | 5.1 (101.9%) | 5.8 (115.3%) | 5.0 (100.7%) | 18.0 | 18.1 |
| SD |  | 1.0 | 1.1 | 0.7 |  |  |
| RSD |  | 19.6 | 19.7 | 13.3 |  |  |
| Mean | 15.0 | 14.7 (97.8%) | 15.3 (102.3%) | 14.4 (96.0%) | 5.4 | 5.9 |
| SD |  | 0.7 | 0.8 | 0.9 |  |  |
| RSD |  | 4.9 | 5.3 | 6.1 |  |  |
| Mean | 50.0 | 52.5 (104.9%) | 49.1 (98.2%) | 50.2 (100.5%) | 4.2 | 5.1 |
| SD |  | 3.0 | 1.0 | 1.8 |  |  |
| RSD |  | 5.7 | 2.0 | 3.6 |  |  |
| Mean | 4500.0 | 5169.7 (114.9%) | 4365.0 (97.0%) | 4543.9 (101.0%) | 7.8 | 11.8 |
| SD |  | 584.4 | 126.7 | 133.1 |  |  |
| RSD |  | 11.3 | 2.9 | 2.9 |  |  |

Within-day and between-day estimates were conducted with 6 independent measurements on three diﬀerent days. Values in parentheses represent accuracy of the method
