# Supplementary material for: A stable isotope dilution tandem mass spectrometry method of major kavalactones and its applications
Source: PLoS One. 2018 May 24;13(5):e0197940. doi: 10.1371/journal.pone.0197940 (PMC5993114; doi:10.1371/journal.pone.0197940)
Supplement: S5 Table — Within-day and between-day estimates were conducted with 6 independent measurements on three different days. Values in parentheses represent accuracy of the method. (DOCX) [file pone.0197940.s010.docx]

**S5 Table. Accuracy, and intraday and interday precision of kavain, DHK, methysticin, DHM and desmethoxyyangonin (pg/*µ*L) in the control mouse serum at spiking level of 0.8, 8, 80 and 8000 pg/mg tissue.**

|  | **Spiked level (pg/*µ*L)** | **Day 1** | **Day 2** | **Day 3** | **Within-day (CV%)** | **Between-day (CV%)** |
| --- | --- | --- | --- | --- | --- | --- |
| **Kavain** | | | | | | |
| Mean | 0.80 | 0.66 (82.8%) | 0.70 (87.7%) | 0.70 (87.4%) | 11.4 | 10.9 |
| SD |  | 0.07 | 0.08 | 0.09 |  |  |
| RSD |  | 10.61 | 10.65 | 12.33 |  |  |
| Mean | 8.0 | 7.9 (99.3%) | 7.3 (91.0%) | 8.4 (104.6%) | 4.2 | 8.0 |
| SD |  | 0.3 | 0.5 | 0.2 |  |  |
| RSD |  | 3.2 | 6.4 | 2.6 |  |  |
| Mean | 80.0 | 89.3 (111.6%) | 90.0 (112.6%) | 88.7 (110.9%) | 2.6 | 2.5 |
| SD |  | 3.3 | 1.1 | 2.1 |  |  |
| RSD |  | 3.7 | 1.2 | 2.4 |  |  |
| Mean | 8000.0 | 7621.2 (95.3%) | 7633.5 (95.4%) | 7657.6 (95.7%) | 2.6 | 2.4 |
| SD |  | 204.8 | 183.4 | 208.4 |  |  |
| RSD |  | 2.7 | 2.4 | 2.7 |  |  |
| **DHK** | | | | | | |
| Mean | 0.80 | 0.70 (88.1%) | 0.65 (81.6%) | 0.71 (88.9%) | 16.1 | 15.4 |
| SD |  | 0.04 | 0.19 | 0.05 |  |  |
| RSD |  | 5.68 | 28.26 | 7.59 |  |  |
| Mean | 8.0 | 7.4 (92.1%) | 7.6 (94.5%) | 7.2 (89.5%) | 5.0 | 5.3 |
| SD |  | 0.4 | 0.3 | 0.4 |  |  |
| RSD |  | 4.9 | 4.4 | 5.8 |  |  |
| Mean | 80.0 | 78.4 (98.0%) | 79.8 (99.7%) | 82.1 (102.6%) | 6.3 | 6.2 |
| SD |  | 8.0 | 3.6 | 1.8 |  |  |
| RSD |  | 10.2 | 4.5 | 2.2 |  |  |
| Mean | 8000.0 | 8428.6 (105.4%) | 8566.8 (107.1%) | 8559.4 (107.0%) | 8.1 | 7.5 |
| SD |  | 1151.3 | 296.7 | 223.0 |  |  |
| RSD |  | 13.7 | 3.5 | 2.6 |  |  |
| **Methysticin** | | | | | | |
| Mean | 0.80 | 0.69 (86.4%) | 0.66 (82.5%) | 0.78 (96.8%) | 16.2 | 17.3 |
| SD |  | 0.07 | 0.12 | 0.13 |  |  |
| RSD |  | 9.57 | 18.73 | 16.43 |  |  |
| Mean | 8.0 | 8.0 (99.8%) | 7.4 (92.5%) | 8.6 (107.4%) | 5.7 | 9.1 |
| SD |  | 0.4 | 0.6 | 0.3 |  |  |
| RSD |  | 4.9 | 8.6 | 2.9 |  |  |
| Mean | 80.0 | 81.1 (101.4%) | 83.0 (103.7%) | 80.8 (101.0%) | 3.0 | 3.1 |
| SD |  | 2.1 | 2.2 | 3.0 |  |  |
| RSD |  | 2.6 | 2.6 | 3.8 |  |  |
| Mean | 8000.0 | 7663.2 (110.9%) | 7738.8 (111.8%) | 7556.0 (111.2%) | 2.9 | 2.7 |
| SD |  | 62.1 | 187.9 | 211.1 |  |  |
| RSD |  | 0.8 | 2.4 | 2.8 |  |  |
| **DHM** | | | | | | |
| Mean | 0.80 | 0.72 (89.5%) | 0.77 (96.5%) | 0.76 (95.3%) | 4.2 | 5.5 |
| SD |  | 0.03 | 0.02 | 0.04 |  |  |
| RSD |  | 4.31 | 3.07 | 5.08 |  |  |
| Mean | 8.0 | 8.4 (104.9%) | 7.9 (98.6%) | 8.6 (107.9%) | 4.9 | 6.4 |
| SD |  | 0.5 | 0.4 | 0.4 |  |  |
| RSD |  | 5.6 | 4.8 | 4.2 |  |  |
| Mean | 80.0 | 82.7 (103.4%) | 81.6 (102.1%) | 85.3 (106.7%) | 3.4 | 3.8 |
| SD |  | 1.2 | 3.4 | 3.3 |  |  |
| RSD |  | 1.5 | 4.1 | 3.9 |  |  |
| Mean | 8000.0 | 8662.0 (108.3%) | 8384.5 (104.8%) | 8542.6 (106.8%) | 2.5 | 2.8 |
| SD |  | 215.0 | 155.1 | 260.9 |  |  |
| RSD |  | 2.5 | 1.9 | 3.1 |  |  |
| **Desmethoxyyangonin** | | | | | | |
| Mean | 0.80 | 0.73 (90.9%) | 0.72 (89.9%) | 0.71 (89.1%) | 3.9 | 3.7 |
| SD |  | 0.03 | 0.03 | 0.02 |  |  |
| RSD |  | 4.03 | 4.46 | 3.10 |  |  |
| Mean | 8.0 | 8.1 (100.8%) | 7.7 (96.8%) | 7.6 (95.4%) | 6.8 | 6.8 |
| SD |  | 0.6 | 0.4 | 0.6 |  |  |
| RSD |  | 7.0 | 5.1 | 7.6 |  |  |
| Mean | 80.0 | 73.4 (91.8%) | 71.0 (88.7%) | 69.8 (87.2%) | 3.2 | 3.9 |
| SD |  | 1.8 | 1.9 | 3.0 |  |  |
| RSD |  | 2.5 | 2.7 | 4.4 |  |  |
| Mean | 8000.0 | 7663.2 (95.8%) | 7738.8 (96.7%) | 7556.0 (94.4%) | 2.2 | 2.3 |
| SD |  | 62.1 | 187.9 | 211.1 |  |  |
| RSD |  | 0.8 | 2.4 | 2.8 |  |  |

Within-day and between-day estimates were conducted with 6 independent measurements on three diﬀerent days. Values in parentheses represent accuracy of the method
