# Supplementary material for: A stable isotope dilution tandem mass spectrometry method of major kavalactones and its applications
Source: PLoS One. 2018 May 24;13(5):e0197940. doi: 10.1371/journal.pone.0197940 (PMC5993114; doi:10.1371/journal.pone.0197940)
Supplement: S6 Table — Within-day and between-day estimates were conducted with 6 independent measurements on three different days. Values in parentheses represent accuracy of the method. (DOCX) [file pone.0197940.s011.docx]

**S6 Table. Accuracy, and intraday and interday precision and reproducibility of kavain, DHK, methysticin, DHM and desmethoxyyangonin (pg/*µ*L) in the plasma of pre-kava human subjects at spiking level of 0.15, 0.4, 1 and 2 pg/*µ*L.**

|  | **Spiked level (pg/*µ*L)** | **Day 1** | **Day 2** | **Day 3** | **Within-day (CV%)** | **Between-day (CV%)** |
| --- | --- | --- | --- | --- | --- | --- |
| **Kavain** | | | | | | |
| Mean | 0.15 | 0.13 (89.1%) | 0.13 (85.5%) | 0.14 (92.4%) | 4.9 | 5.9 |
| SD |  | 0.01 | 0.01 | 0.01 |  |  |
| RSD |  | 5.12 | 4.88 | 5.11 |  |  |
| Mean | 0.40 | 0.38 (94.6%) | 0.38 (94.3%) | 0.37 (91.4%) | 4.7 | 4.7 |
| SD |  | 0.02 | 0.01 | 0.02 |  |  |
| RSD |  | 4.48 | 3.78 | 5.69 |  |  |
| Mean | 1.00 | 0.96 (96.3%) | 0.98 (98.0%) | 1.04 (104.4%) | 3.9 | 5.6 |
| SD |  | 0.04 | 0.03 | 0.05 |  |  |
| RSD |  | 3.72 | 3.50 | 4.37 |  |  |
| Mean | 2.00 | 1.94 (97.0%) | 2.06 (103.1%) | 2.04 (101.9%) | 2.1 | 3.7 |
| SD |  | 0.05 | 0.04 | 0.04 |  |  |
| RSD |  | 2.42 | 1.90 | 1.91 |  |  |
| **DHK** | | | | | | |
| Mean | 0.15 | 0.13 (86.6%) | 0.12 (82.8%) | 0.14 (97.1%) | 4.5 | 9.4 |
| SD |  | 0.00 | 0.01 | 0.01 |  |  |
| RSD |  | 2.92 | 5.03 | 3.49 |  |  |
| Mean | 0.40 | 0.39 (97.3%) | 0.39 (96.8%) | 0.38 (94.8%) | 8.1 | 7.6 |
| SD |  | 0.01 | 0.04 | 0.04 |  |  |
| RSD |  | 2.19 | 10.00 | 9.79 |  |  |
| Mean | 1.00 | 0.87 (86.9%) | 0.91 (91.0%) | 1.03 (102.8%) | 6.4 | 10.7 |
| SD |  | 0.04 | 0.02 | 0.09 |  |  |
| RSD |  | 4.93 | 2.43 | 8.78 |  |  |
| Mean | 2.00 | 2.02 (100.8%) | 1.86 (92.8%) | 1.91 (95.6%) | 5.6 | 6.7 |
| SD |  | 0.11 | 0.11 | 0.11 |  |  |
| RSD |  | 5.53 | 5.72 | 5.73 |  |  |
| **Methysticin** | | | | | | |
| Mean | 0.15 | 0.14 (90.7%) | 0.13 (88.9%) | 0.14 (90.5%) | 6.1 | 5.7 |
| SD |  | 0.01 | 0.00 | 0.01 |  |  |
| RSD |  | 9.59 | 2.60 | 3.49 |  |  |
| Mean | 0.40 | 0.38 (95.1%) | 0.38 (94.7%) | 0.39 (96.6%) | 2.6 | 2.6 |
| SD |  | 0.00 | 0.01 | 0.01 |  |  |
| RSD |  | 1.16 | 2.82 | 3.29 |  |  |
| Mean | 1.00 | 0.96 (96.0%) | 0.94 (93.9%) | 1.04 (103.6%) | 4.4 | 6.7 |
| SD |  | 0.04 | 0.03 | 0.05 |  |  |
| RSD |  | 4.51 | 2.66 | 4.92 |  |  |
| Mean | 2.00 | 2.04 (102.2%) | 1.96 (98.0%) | 2.04 (101.9%) | 2.6 | 3.3 |
| SD |  | 0.05 | 0.02 | 0.07 |  |  |
| RSD |  | 2.23 | 1.06 | 3.61 |  |  |
| **DHM** | | | | | | |
| Mean | 0.15 | 0.13 (89.2%) | 0.14 (93.5%) | 0.14 (96.2%) | 3.2 | 4.8 |
| SD |  | 0.002 | 0.004 | 0.01 |  |  |
| RSD |  | 1.52 | 2.99 | 4.30 |  |  |
| Mean | 0.40 | 0.38 (94.9%) | 0.38 (95.9%) | 0.37 (91.3%) | 3.2 | 3.9 |
| SD |  | 0.01 | 0.01 | 0.01 |  |  |
| RSD |  | 3.63 | 3.36 | 2.31 |  |  |
| Mean | 1.00 | 0.99 (98.9%) | 0.99 (99.2%) | 1.01 (100.5%) | 4.8 | 4.5 |
| SD |  | 0.06 | 0.04 | 0.05 |  |  |
| RSD |  | 5.57 | 4.29 | 4.56 |  |  |
| Mean | 2.00 | 2.20 (99.7%) | 2.02 (101.2%) | 2.04 (101.8%) | 2.2 | 2.3 |
| SD |  | 0.05 | 0.04 | 0.03 |  |  |
| RSD |  | 2.72 | 2.19 | 1.68 |  |  |
| **Desmethoxyyangonin** | | | | | | |
| Mean | 0.15 | 0.13 (83.6%) | 0.13 (89.2%) | 0.13 (86.7%) | 5.4 | 5.9 |
| SD |  | 0.01 | 0.01 | 0.01 |  |  |
| RSD |  | 5.89 | 4.68 | 5.65 |  |  |
| Mean | 0.40 | 0.39 (98.2%) | 0.37 (92.3%) | 0.36 (89.9%) | 5.4 | 6.8 |
| SD |  | 0.02 | 0.01 | 0.02 |  |  |
| RSD |  | 5.75 | 1.61 | 7.2 |  |  |
| Mean | 1.00 | 0.98 (97.6%) | 0.93 (93.1%) | 0.94 (94.3%) | 8.8 | 8.4 |
| SD |  | 0.13 | 0.05 | 0.03 |  |  |
| RSD |  | 13.48 | 5.84 | 2.99 |  |  |
| Mean | 2.00 | 2.06 (102.9%) | 2.01 (100.6%) | 2.03 (101.3%) | 3.3 | 3.2 |
| SD |  | 0.05 | 0.04 | 0.01 |  |  |
| RSD |  | 2.47 | 1.82 | 4.78 |  |  |

Within-day and between-day estimates were conducted with 6 independent measurements on three diﬀerent days. Values in parentheses represent accuracy of the method
