# Supplementary material for: A stable isotope dilution tandem mass spectrometry method of major kavalactones and its applications
Source: PLoS One. 2018 May 24;13(5):e0197940. doi: 10.1371/journal.pone.0197940 (PMC5993114; doi:10.1371/journal.pone.0197940)
Supplement: S7 Table — Within-day and between-day estimates were conducted with 6 independent measurements on three different days. Values in parentheses represent accuracy of the method. (DOCX) [file pone.0197940.s012.docx]

S7 Table. Accuracy, and intraday and interday precision of kavain, DHK, methysticin, DHM and desmethoxyyangonin (pg/*µ*L) in the urine of pre-kava human subjects at spiking level of 0.15, 0.4, 1 and 2 pg/*µ*L.

|  | **Spiked level (pg/*µ*L)** | **Day 1** | **Day 2** | **Day 3** | **Within-day (CV%)** | **Between-day (CV%)** |
| --- | --- | --- | --- | --- | --- | --- |
| **Kavain** | | | | | | |
| Mean | 0.15 | 0.14 (90.2%) | 0.14 (90.4%) | 0.15 (98.0%) | 4.9 | 6.6 |
| SD |  | 0.01 | 0.01 | 0.00 |  |  |
| RSD |  | 7.08 | 4.51 | 2.35 |  |  |
| Mean | 0.40 | 0.37 (93.2%) | 0.40 (100.2%) | 0.38 (94.7%) | 4.3 | 5.5 |
| SD |  | 0.02 | 0.02 | 0.01 |  |  |
| RSD |  | 5.54 | 3.81 | 3.43 |  |  |
| Mean | 1.00 | 0.99 (99.0%) | 1.01 (100.9%) | 0.96 (95.7%) | 3.5 | 4.2 |
| SD |  | 0.04 | 0.02 | 0.04 |  |  |
| RSD |  | 3.82 | 2.41 | 4.05 |  |  |
| Mean | 2.00 | 1.95 (97.2%) | 2.04 (101.9%) | 2.04 (102.0%) | 3.0 | 3.8 |
| SD |  | 0.06 | 0.07 | 0.05 |  |  |
| RSD |  | 2.95 | 3.60 | 2.23 |  |  |
| **DHK** | | | | | | |
| Mean | 0.15 | 0.13 (86.4%) | 0.12 (80.1%) | 0.15 (99.9%) | 5.5 | 12.6 |
| SD |  | 0.01 | 0.01 | 0.01 |  |  |
| RSD |  | 3.85 | 5.23 | 6.37 |  |  |
| Mean | 0.40 | 0.39 (96.9%) | 0.40 (99.8%) | 0.41 (101.7%) | 10.3 | 9.8 |
| SD |  | 0.01 | 0.05 | 0.04 |  |  |
| RSD |  | 2.69 | 13.15 | 10.45 |  |  |
| Mean | 1.00 | 0.99 (99.4%) | 1.09 (109.4%) | 1.03 (103.0%) | 11.6 | 11.7 |
| SD |  | 0.03 | 0.19 | 0.08 |  |  |
| RSD |  | 3.07 | 17.07 | 7.48 |  |  |
| Mean | 2.00 | 2.03 (101.3%) | 1.90 (95.0%) | 1.86 (93.0%) | 6.5 | 7.4 |
| SD |  | 0.09 | 0.07 | 0.19 |  |  |
| RSD |  | 4.38 | 3.53 | 10.06 |  |  |
| **Methysticin** | | | | | | |
| Mean | 0.15 | 0.15 (100.3%) | 0.14 (92.4%) | 0.14 (92.6%) | 7.1 | 8.1 |
| SD |  | 0.01 | 0.01 | 0.01 |  |  |
| RSD |  | 6.44 | 4.28 | 9.71 |  |  |
| Mean | 0.40 | 0.38 (94.3%) | 0.40 (99.1%) | 0.39 (98.0%) | 2.6 | 3.6 |
| SD |  | 0.00 | 0.01 | 0.01 |  |  |
| RSD |  | 0.95 | 2.97 | 3.12 |  |  |
| Mean | 1.00 | 1.01 (101.1%) | 1.04 (104.0%) | 0.97 (96.6%) | 2.2 | 4.1 |
| SD |  | 0.03 | 0.01 | 0.03 |  |  |
| RSD |  | 2.51 | 1.26 | 2.58 |  |  |
| Mean | 2.00 | 2.01 (101.0%) | 1.99 (96.8%) | 1.98 (100.1%) | 2.6 | 3.2 |
| SD |  | 0.07 | 0.07 | 0.06 |  |  |
| RSD |  | 3.44 | 3.42 | 3.21 |  |  |
| **DHM** | | | | | | |
| Mean | 0.15 | 0.14 (95.0%) | 0.14 (94.1%) | 0.15 (98.6%) | 3.5 | 4.1 |
| SD |  | 0.003 | 0.006 | 0.005 |  |  |
| RSD |  | 2.37 | 4.53 | 3.38 |  |  |
| Mean | 0.40 | 0.38 (93.8%) | 0.38 (99.2%) | 0.38 (94.3%) | 4.6 | 5.2 |
| SD |  | 0.01 | 0.02 | 0.02 |  |  |
| RSD |  | 3.21 | 5.75 | 3.96 |  |  |
| Mean | 1.00 | 1.02 (101.7%) | 1.01 (101.0%) | 0.93 (93.1%) | 3.1 | 5.5 |
| SD |  | 0.03 | 0.02 | 0.04 |  |  |
| RSD |  | 3.29 | 2.06 | 3.97 |  |  |
| Mean | 2.00 | 2.02 (101.2%) | 2.02 (100.9%) | 2.00 (99.8%) | 2.6 | 2.5 |
| SD |  | 0.06 | 0.06 | 0.02 |  |  |
| RSD |  | 3.05 | 3.06 | 1.08 |  |  |
| **Desmethoxyyangonin** | | | | | | |
| Mean | 0.15 | 0.13 (85.4%) | 0.14 (90.7%) | 0.13 (88.8%) | 4.6 | 5.2 |
| SD |  | 0.01 | 0.01 | 0.004 |  |  |
| RSD |  | 6.47 | 3.77 | 3.01 |  |  |
| Mean | 0.40 | 0.39 (97.5%) | 0.38 (94.2%) | 0.36 (90.8%) | 4.9 | 5.7 |
| SD |  | 0.03 | 0.01 | 0.01 |  |  |
| RSD |  | 6.89 | 3.54 | 2.58 |  |  |
| Mean | 1.00 | 1.03 (102.6%) | 1.01 (101.2%) | 0.95 (94.6%) | 4.4 | 5.9 |
| SD |  | 0.06 | 0.03 | 0.04 |  |  |
| RSD |  | 6.01 | 2.86 | 3.76 |  |  |
| Mean | 2.00 | 2.01 (100.3%) | 1.99 (99.4%) | 1.98 (98.8%) | 3.4 | 3.2 |
| SD |  | 0.07 | 0.07 | 0.06 |  |  |
| RSD |  | 3.44 | 3.42 | 3.21 |  |  |

Within-day and between-day estimates were conducted with 6 independent measurements on three diﬀerent days. Values in parentheses represent accuracy of the method
