# Supplementary material for: A stable isotope dilution tandem mass spectrometry method of major kavalactones and its applications
Source: PLoS One. 2018 May 24;13(5):e0197940. doi: 10.1371/journal.pone.0197940 (PMC5993114; doi:10.1371/journal.pone.0197940)
Supplement: S8 Table — The value in parentheses is the relative amount of individual kavalactone to the total kavalactones. (DOCX) [file pone.0197940.s013.docx]

**S8 Table. The composition of two kava products.**

|  | **Kavain** | **DHK** | **Methysticin** | **DHM** | **Desmethoxyyangonin** |
| --- | --- | --- | --- | --- | --- |
| **Ethanolic kava extract** | | | | | |
| Composition  (g/ g kava extract) | 0.172 ± 0.030  (27.2%) | 0.247 ± 0.018  (39.1%) | 0.021 ± 0.009  (3.4%) | 0.089 ± 0.008  (14.1%) | 0.103 ± 0.011  (16.2%) |
| **Soft-gel kava capsule** | | | | | |
| Composition  (mg/soft gel capsule) | 0.026 ± 0.001  (33.7%) | 0.014 ± 0.001  (18.2%) | 0.008 ± 0.001  (10.4%) | 0.021 ± 0.002  (27.3%) | 0.008 ± 0.001  (10.4%) |

The value in parentheses is the ratio of the mean value of the amount of individual kavalactone to the total kavalactones. (Mean ± SD, n=3).
