# Supplementary material for: A stable isotope dilution tandem mass spectrometry method of major kavalactones and its applications
Source: PLoS One. 2018 May 24;13(5):e0197940. doi: 10.1371/journal.pone.0197940 (PMC5993114; doi:10.1371/journal.pone.0197940)
Supplement: S9 Table — Within-day and between-day estimates were conducted with three independent measurements on three different days. (DOCX) [file pone.0197940.s014.docx]

**S9 Table. Within-day and between-day estimates of kavain, dihydrokavain, methysticin, DHM and desmethoxyyangonin in the 1.5-h mouse liver tissues.**

| **Mice** |  | **Day 1**  **(pg/mg tissue)** | **Day 2**  **(pg/mg tissue)** | **Day 3**  **(pg/mg tissue)** | **CV (%)**  **Within-day** | **CV (%)**  **Between-day** |
| --- | --- | --- | --- | --- | --- | --- |
| DHM | Mean | 7060 | 6418 | 7192 | 6.8 | 8.7 |
|  | SD | 85 | 306 | 748 |  |  |
|  | RSD | 1.2 | 4.8 | 10.4 |  |  |
| DHK | Mean | 13099 | 15810 | 13646 | 6.0 | 11.7 |
|  | SD | 890 | 950 | 638 |  |  |
|  | RSD | 6.8 | 6.0 | 4.7 |  |  |
| Kavain | Mean | 22928 | 23650 | 26663 | 4.4 | 9.0 |
|  | SD | 704 | 1674 | 467 |  |  |
|  | RSD | 3.1 | 7.1 | 1.8 |  |  |
| Methysticin | Mean | 3455 | 3146 | 3475 | 5.3 | 7.3 |
|  | SD | 181 | 152 | 192 |  |  |
|  | RSD | 5.2 | 4.8 | 5.5 |  |  |
| Desmethoxyyangonin | Mean | 5246 | 4099 | 4700 | 7.2 | 14.5 |
|  | SD | 286 | 113 | 465 |  |  |
|  | RSD | 5.4 | 2.8 | 9.9 |  |  |

Within-day and between-day estimates were conducted with three independent measurements on three diﬀerent days
