# Supplementary material for: A stable isotope dilution tandem mass spectrometry method of major kavalactones and its applications
Source: PLoS One. 2018 May 24;13(5):e0197940. doi: 10.1371/journal.pone.0197940 (PMC5993114; doi:10.1371/journal.pone.0197940)
Supplement: S10 Table — (DOCX) [file pone.0197940.s015.docx]

**S10 Table. Pharmacokinetic parameters of kavain, DHK, methysticin, DHM and desmethoxyyangonin.**

| A. Pharmacokinetic parameters of kavain | | | | |
| --- | --- | --- | --- | --- |
| Parameters | Serum | Liver | Lung | Brain |
| C_max_ (ng/mL or ng/g) | 744.3±112.4 | 15522.3±3913.5 | 3107.5±447.4 | 2060.0±652.7 |
| T_max_ (h) | 1.5 | 0.5 | 1.5 | 1.5 |
| AUC_0-t_ (h*ng/ml or h*ng/g) | 2883.9 | 33143.9 | 9568.5 | 6475.6 |
| MRT (h) | 6.8 | 2.0 | 5.5 | 5.6 |
| Cl/F (L/h/kg or kg/h/kg) | 1.37 | 0.20 | 0.50 | 0.64 |

| B. Pharmacokinetic parameters of DHK | | | | |
| --- | --- | --- | --- | --- |
| Parameters | Serum | Liver | Lung | Brain |
| C_max_ (ng/mL or ng/g) | 983.1±206.6 | 9327.0±2073.4 | 2387.5±73.9 | 1224.9±311.5 |
| T_max_ (h) | 1.5 | 0.5 | 1.5 | 1.5 |
| AUC_0-t_ (h*ng/ml or h*ng/g) | 7689.8 | 25143.4 | 7799.7 | 4954.4 |
| MRT (h) | 9.0 | 2.6 | 5.3 | 7.2 |
| Cl/F (L/h/kg or kg/h/kg) | 0.56 | 0.38 | 0.66 | 0.39 |

| C. Pharmacokinetic parameters of methysticin | | | | |
| --- | --- | --- | --- | --- |
| Parameters | Serum | Liver | Lung | Brain |
| C_max_ (ng/mL or ng/g) | 501.9±40.9 | 2482.0±696.1 | 562.8±82.5 | 362.3±121.1 |
| T_max_ (h) | 1.5 | 1.5 | 1.5 | 1.5 |
| AUC_0-t_ (h*ng/ml or h*ng/g) | 1723.9 | 7046.6 | 2627.3 | 2120.0 |
| MRT (h) | 5.8 | 2.5 | 7.8 | 8.0 |
| Cl/F (L/h/kg or kg/h/kg) | 0.31 | 0.11 | 0.14 | 0.24 |

| D. Pharmacokinetic parameters of DHM | | | | |
| --- | --- | --- | --- | --- |
| Parameters | Serum | Liver | Lung | Brain |
| C_max_ (ng/mL or ng/g) | 747.2±141.1 | 4142.0±1466.7 | 1077.9±69.9 | 540.9±149.6 |
| T_max_ (h) | 1.5 | 0.5 | 1.5 | 1.5 |
| AUC_0-t_ (h*ng/ml or h*ng/g) | 7534.2 | 10392.6 | 3870.3 | 3277.3 |
| MRT (h) | 10.8 | 3.2 | 6.0 | 9.2 |
| Cl/F (L/h/kg or kg/h/kg) | 0.14 | 0.31 | 0.73 | 0.23 |

| E. Pharmacokinetic parameters of desmethoxyyangonin | | | | |
| --- | --- | --- | --- | --- |
| Parameters | Serum | Liver | Lung | Brain |
| C_max_ (ng/mL or ng/g) | 64.4±23.5 | 3148.3±1073.5 | 326.5±85.2 | 269.9±118.9 |
| T_max_ (h) | 1.5 | 0.5 | 1.5 | 1.5 |
| AUC_0-t_ (h*ng/ml or h*ng/g) | - | 7123.8 | 1314.7 | 1249.3 |
| MRT (h) | - | 1.9 | 6.0 | 8.2 |
| Cl/F (L/h/kg or kg/h/kg) | - | 0.56 | 1.18 | 0.66 |

^a^Each value represents the average of three mice dosed orally at 40 mg/kg of kavakava extract containing equivalent dose of 6.9 mg/kg dose of Kavain; 9.9 mg/kg dose of dihydrokavain; 0.84 mg/kg dose of methysticin; 3.6 mg/kg dose of dihydromethysticin; and 4.1 mg/kg dose of desmethoxyyangonin. Values of C_max_ are mean ± SEM. *Abbreviations:* AUC_0-t_ = area under the serum concentration-time curve upto last sampling time, C_max_ = serum peak concentration, MRT = mean residence time, t_max_ = time to C_max_, Cl/F = clearance.
